# Psychological Treatment of Depression in People Aged 65 Years and Over: A Systematic Review of Efficacy, Safety, and Cost-Effectiveness

**DOI:** 10.1371/journal.pone.0160859

**Published:** 2016-08-18

**Authors:** Ulf Jonsson, Göran Bertilsson, Per Allard, Harald Gyllensvärd, Anne Söderlund, Anne Tham, Gerhard Andersson

**Affiliations:** 1 Department of Clinical Neuroscience, Division of Insurance Medicine, Karolinska Institutet, Stockholm, Sweden; 2 Department of Neuroscience, Child and adolescent psychiatry, Uppsala University, Uppsala, Sweden; 3 National Board of Health and Welfare, Stockholm, Sweden; 4 Swedish Agency for Health Technology Assessment and Assessment of Social Services, Stockholm, Sweden; 5 Department of Clinical Sciences, Division of Psychiatry, Umeå University, Umeå, Sweden; 6 Department of Medical and Health Sciences, Linköping University, Linköping, Sweden; 7 School of Health, Care and Social Welfare, Physiotherapy, Mälardalen University, Mälardalen, Våsterås, Sweden; 8 Department of Clinical Neuroscience, Psychiatry Section, Karolinska Institutet, Stockholm, Sweden; 9 Department of Behavioural Sciences and Learning, Linköping University, Linköping, Sweden; Istituto Superiore Di Sanita, ITALY

## Abstract

**Objectives:**

Depression in elderly people is a major public health concern. As response to antidepressants is often unsatisfactory in this age group, there is a need for evidence-based non-pharmacological treatment options. Our objectives were twofold: firstly, to synthesize published trials evaluating efficacy, safety and cost-effectiveness of psychological treatment of depression in the elderly and secondly, to assess the quality of evidence.

**Method:**

The electronic databases PubMed, EMBASE, Cochrane Library, CINAL, Scopus, and PsycINFO were searched up to 23 May 2016 for randomized controlled trials (RCTs) of psychological treatment for depressive disorders or depressive symptoms in people aged 65 years and over. Two reviewers independently assessed relevant studies for risk of bias. Where appropriate, the results were synthesized in meta-analyses. The quality of the evidence was graded according to GRADE (Grading of Recommendations Assessment, Development and Evaluation).

**Results:**

Twenty-two relevant RCTs were identified, eight of which were excluded from the synthesis due to a high risk of bias. Of the remaining trials, six evaluated problem-solving therapy (PST), five evaluated other forms of cognitive behavioural therapy (CBT), and three evaluated life review/reminiscence therapy. In frail elderly with depressive symptoms, the evidence supported the efficacy of PST, with large but heterogeneous effect sizes compared with treatment as usual. The results for life-review/reminiscence therapy and CBT were also promising, but because of the limited number of trials the quality of evidence was rated as very low. Safety data were not reported in any included trial. The only identified cost-effectiveness study estimated an incremental cost per additional point reduction in Beck Depression Inventory II score for CBT compared with talking control and treatment as usual.

**Conclusion:**

Psychological treatment is a feasible option for frail elderly with depressive symptoms. However, important questions about efficacy, generalizability, safety and cost-effectiveness remain.

## Introduction

Depression is a major public health problem and a leading cause of disability [1]. A study of community samples from nine European centres reported a prevalence of 12.3% for depressive disorder among people aged 65 years and older, and a cross-centre prevalence of 26–40% for depressive mood [2]. With a growing elderly population in many countries, late-life depression is an increasing challenge. Health care costs have been estimated to be about twice as high or more for depressed as for non-depressed older adults and informal care costs are about four times as high [3–5], underscoring the need for treatments that are effective, safe and cost-effective.

Many elderly patients have unsatisfactory responses to antidepressants [6], including problematic side effects and the risk of drug interactions. Hence there is a need for evidence-based non-pharmacological treatment options. In working age adults with mild to moderate depression, psychological treatment is effective [7]. However, in older adults depression may differ in terms of biological, psychological and social characteristics [8]. General medical conditions, cognitive deficits, and other deficits associated with old age might decrease the applicability of some psychological treatments, limiting the generalizability of outcomes reported for younger adults or depressed but otherwise healthy elderly. Moreover, loss of significant others and friends and changes in role function and social participation associated with retirement and old age, could have an impact on treatment outcome.

Several meta-analyses and systematic reviews have been published on the effects of psychological treatment for older adults [9–11]. In a recent review, Cuijpers and colleagues [9] included 44 studies in which different psychotherapies were compared with waitlist, other forms of psychotherapy or pharmacotherapy. The overall effect size was *g* = 0.64, in favour of psychotherapy. The strongest evidence was found for cognitive behavioural therapy (CBT) and problem-solving therapy (PST). A recent review by Simon and colleagues suggested that older adults with depression and cognitive deficits could benefit from CBT [11].

The present systematic review expands on currently available knowledge. While several of the studies included in previous reviews recruited participants from 50 or 55 years of age, the present review was limited exclusively to studies in which all participants were aged 65 years or over, i.e. in accordance with the Organisation for Economic Co-operation and Development (OECD) definition of an elderly population. In several OECD countries the retirement age is 65, and in some countries it has recently been raised to 67 or more [12]. In many OECD regions more than 20% of the population is aged 65 or older. A specific focus on this age group is therefore warranted. The review was not restricted to one therapy form or format, and in contrast to previous systematic reviews any available safety data (e.g., adverse events) were to be coded and available cost-effectiveness studies were to be reviewed.

Another feature distinguishing this review from previous reviews was that information of relevance to the generalizability of the results was extracted, with special reference to the participants’ level of frailty. This is likely to be a key source of variance in treatment outcome for depressed elderly, making generalizability across age groups unclear. Two principal models of frailty have emerged: the phenotype model and the cumulative deficit model [13]. The frailty phenotype is characterized by unintentional weight loss, self-reported exhaustion, low energy expenditure, slow gait speed, and weak grip strength. According to the cumulative deficit model, on the other hand, frailty is defined as the cumulative effect of individual deficits. Signs of frailty in the study participants are likely to be reported in a variety of ways across trials. We therefore screened the reports for any indicator of frailty according to either one of these models.

The objectives were twofold: firstly, to synthesize published trials evaluating efficacy, safety and cost-effectiveness of psychological treatment of depression in the elderly and secondly, to assess the quality of evidence. Initially a systematic review was conducted of randomized controlled trials of psychological treatment for depressive disorders or depressive symptoms in people aged 65 years and over, compared with alternative treatments or no treatment. The quality of the evidence was then graded according to the international system GRADE (Grading of Recommendations Assessment, Development and Evaluation) [14].

## Method

### Protocol and registration

This systematic review was part of a project investigating the efficacy, safety, and cost-effectiveness of treatment of depression in the elderly, conducted within the framework of the Swedish Agency for Health Technology Assessment and Assessment of Social Services, SBU (www.sbu.se/en/), a public agency which conducts health technology assessments. Methods of analysis and inclusion criteria for the project were specified in advance, as a part of the internal process at SBU. No protocol has been published.

### Eligibility criteria

The criteria for eligibility included the following characteristics:

#### Population

All participants had to be 65 years or older and either be formally diagnosed with a depressive disorder in accordance with the definitions by American Psychiatric Association and the World Health Organization, or have significant depressive symptoms as measured with a validated scale. Studies explicitly including individuals with bipolar disorders were excluded.

#### Interventions

Any psychological treatment, defined as an intervention based on an explicit psychological theory. Combined treatments or treatment programs (e.g., stepped-care or combined pharmacological and psychological treatment) were not considered, unless the psychological treatment was evaluated separately.

#### Comparator

Any comparator (e.g., any alternative treatment, waitlist, or placebo).

#### Outcome and measures

Change in depressive symptoms or remission, suicidal behaviour, adverse effects, quality of life (QoL), and costs. Any validated measure was acceptable.

#### Study design

Randomized controlled trial (RCT).

#### Setting

Any setting.

#### Language

Studies published in English.

#### Publication type

Studies published in peer-reviewed journals.

### Information sources

Studies were identified by searching electronic databases and by scanning the reference lists of studies meeting the eligibility criteria and relevant systematic reviews. The electronic databases PubMed, EMBASE, Cochrane Library, CINAL, Scopus and PsycINFO were searched up to 23 May 2016.

### Search strategy

Electronic searches were conducted using a combination of medical subject headings (MeSH) and relevant text word terms related to old age, depression and randomized trials. To ensure the sensitivity of the search, a separate search that was not limited to RCTs was conducted for cost-effectiveness studies. (For detailed information about the search strategies, see S1 Appendix.)

### Study selection

Two reviewers independently screened the titles and abstracts for eligibility. All publications of potential relevance according to the inclusion criteria were retrieved in full text. Eligibility for inclusion was independently assessed by two reviewers. Disagreements were resolved by consensus. Reference lists of studies meeting the eligibility criteria and of relevant systematic reviews were screened for additional relevant studies.

### Data collection process

Data were extracted from each included study and inserted into a table by one reviewer. A second reviewer audited the data extraction. Any disagreements were resolved by discussion. If vital information was missing from the published article, provision was made to contact the authors. The authors were ultimately contacted only in one case, in order to clarify if two separate articles reported on the same trial or not.

### Data items

The following information was extracted from the included trials: (1) included population (number randomized, mean age, sex, type of depression, frailty [defined as any indicator of deficits]); (2) treatment (including intensity, duration, delivery, and therapist training); (3) type of comparator (4) outcome and measures; (5) adverse events or deterioration; (6) costs.

### Risk of bias in individual studies

To determine the internal validity of the eligible trials, a pair of reviewers independently assessed the risk of bias according to the SBU checklist. The checklist is based on the CONSORT statement and discloses risk of bias related to six main aspects: selection; treatment (including blinding); measurement; attrition; reporting; conflicts of interest [15]. The checklist was used to reveal shortcomings of the studies. The reviewers thereafter made an assessment of the extent to which the internal validity of the results could have been affected by these shortcomings. A rating of low, moderate or high risk of bias was given to each category of items. Based on the severity of the combined threats to internal validity, an overall rating of risk of bias was then given to each study. Due to the inherently subjective nature of such an assessment, the principal sources of bias on which the overall ratings were based are presented in the results section. Only studies with a low or moderate overall risk of bias were included in the synthesis.

### Planned methods of analysis

The software Review Manager (RevMan) Version 5.3.4 was used for the meta-analyses. Random effects models were applied, due to the substantial heterogeneity that can be expected regarding populations, interventions, comparators and outcome measures across studies. The principal summary measure was the standardized mean difference (Hedge’s *g*), based on the groups’ sample sizes, means and standard deviations for the final follow-up assessment. If more than one assessment point was available, sensitivity analyses including data from previous assessments were conducted. If the number of participants at follow-up was not explicitly stated, we assumed that the group sizes were the same as at randomization.

Studies on subjects with a confirmed depressive disorder were analysed separately from those on subjects with depressive symptoms but no confirmed depressive disorder. The results of studies with interventions or comparators deemed to be too heterogeneous (e.g., due to mode of delivery, components, duration and intensity) were not synthesized. Inconsistencies and heterogeneity disclosed by the meta-analyses were considered when the quality of evidence across studies was assessed.

### Assessing quality of evidence across studies using GRADE

The international system GRADE [14] was used to assess the quality of evidence for efficacy, safety, and cost-effectiveness across studies according to the following four levels:

High quality (⊕⊕⊕⊕) –We are very confident that the true effect lies close to that of the estimate of the effect.

Moderate quality (⊕⊕⊕○) –We are moderately confident in the effect estimate: the true effect is likely to be close to the estimate of the effect, but there is a possibility that it is substantially different.

Low quality (⊕⊕○○) – Our confidence in the effect estimate is limited: the true effect may be substantially different from the estimate of the effect.

Very low quality (⊕○○○) –We have very little confidence in the effect estimate: the true effect is likely to be substantially different from the estimate of the effect.

Under the GRADE system, evidence based on RCTs is initially assessed as high quality, but can be downgraded for reasons such as risk of bias, inconsistency, indirectness, imprecision and publication bias. With the exception of large multi-center trials, SBU routinely grades the evidence as very low when it is based on only one single study. The rating of quality of evidence was guided by the available GRADE literature, and decided through consensus among the authors. The process was audited at the agency by an internal quality and priority group as well as an external council of medical experts.

## Results

### Eligible studies

**Fig 1:**
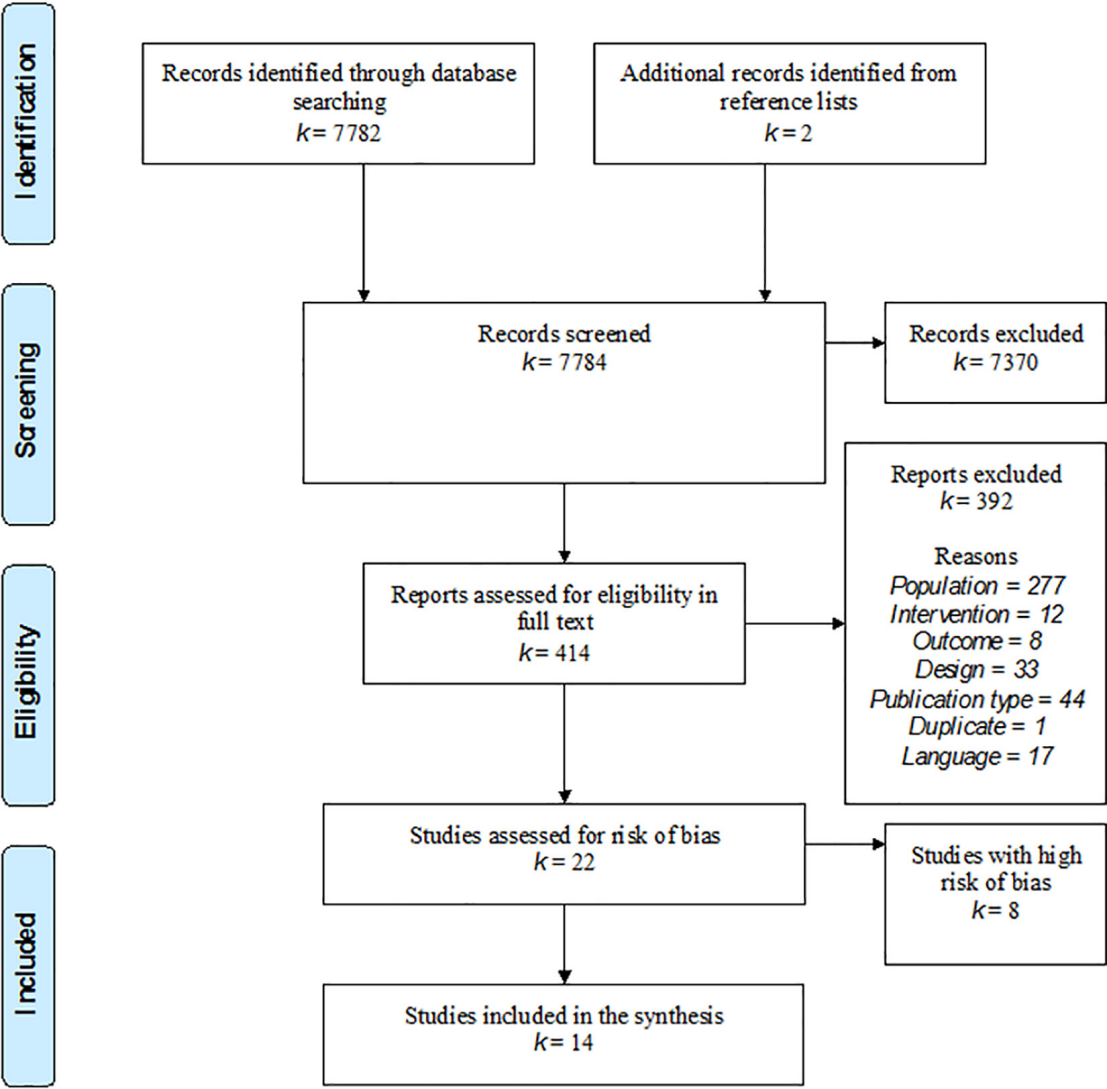
PRISMA flow-chart.

The search yielded a total of 7 784 citations: after review of the abstracts, 7 370 were discarded. The full text of a total of 414 citations was examined, including two identified from reference lists: 392 were excluded as irrelevant (see S2 Appendix for reasons), leaving 22 relevant RCTs (Fig 1).

The literature search for cost-effectiveness studies yielded 1 687 citations. After screening the abstracts, 1 601 citations were excluded and 86 studies were assessed in full text. One study evaluating CBT [16] met the inclusion criteria (see S2 Appendix for reasons for exclusion).

### Risk of bias in individual studies

We identified three principal threats to internal validity, which were judged to be of high relevant for this particular body of evidence: First, several trials had high (≥ 30%) attrition rates in at least one of the treatment groups, which presumably is related to characteristics of the study population. Second, failure to include an active comparator could undermine the internal validity of unblinded trials. Blinding of the patients and the therapists is naturally difficult to attain, while blinded outcome assessment is only partly convincing due to the large subjective component of depressive symptoms. We judged that a credible active comparator has the potential to mitigate the effect of these limitations. Third, several trials included a low number of participants, increasing the risk that important variables (measured and unmeasured) are not kept constant.

The risk of bias, mainly due to high rates of attrition, was deemed to be high in eight of the relevant trials, which were excluded from the synthesis. Four of these trials evaluated various forms of CBT: CBT-based group interventions [17, 18], group-based Competitive Memory Training (COMET) for depressive rumination [19], and a behavioural activation intervention (BE-ACTIV) with individual sessions and staff training [20]. The trials were generally small pilot studies with high rates of attrition. The COMET study, however, was en a relatively large study based on 93 randomized participants. In this study the attrition rate was negligible in the intervention group, while 50% of the control group withdrew consent. Further, a pilot study of PST for residents of long-term care facilities with subsyndromal depression [21] was excluded. Of the 21 participants allocated to the intervention, 14 were lost to follow-up. The authors state that implementation proved to be difficult in terms of study recruitment, intervention acceptance, and compliance. Two of the excluded studies evaluated life review compared to no treatment [22] or supportive therapy [23]. The samples were small and comprised elderly with major depressive disorder (MDD) [23] or clinically significant depressive symptoms [22]. One of these trials was excluded because the trial report was too brief to allow proper assessment [24]. Finally, s study evaluating a therapy called self-worth therapy was excluded due to high attrition rates [24]. While the excluded studies overall indicated positive effects of the interventions, our confidence in these estimates is very low.

The remaining 14 trials were included in the synthesis. Six evaluated PST or interventions in which problem solving was a major component [25–30], five evaluated other forms of CBT [31–35] and three evaluated reminiscence therapy/life review [36–38]. The risk of bias was assessed as low in two of the included trials with low to moderate attrition, active comparators and a relatively large sample sizes [30, 35]. The reaming 12 trials were judged to have moderate risk of bias either due to small samples (< 30 participants in each group) or the use of waitlist or treatment as usual as comparator (Tables 1 and 2).

**Table 1:** Characteristics of the included population in randomized controlled trials of psychological treatment for depression in people aged 65 years and over.

*Intervention* Author, year, country	N, intervention/ control^a^	Drop-out, %, intervention/ control^a^	Age, mean yrs, intervention/ control^a^	Female, %, intervention/ control^a^	Depression criteria	Indicators of frailty^b^
*Problem-solving therapy*						
Alexopolous et al., 2003, USA [25]	12/13	8/15	74	52	MDD; HAM-D≥18	Impaired executive functions
Gellis et al., 2007, USA [28]	24/24	17/17	80	85	CES-D≥22	Medically ill home care patients
Gellis et al., 2010, USA [26]	19/19	5/5	76	95/89	Subthreshold; CES-D≥22	Cardiovascular disease
Gellis et al., 2014, USA [27]	57/58	17/19	78/80	69/63	PHQ-2≥3	Heart failure or COPD
Kiosses et al., 2010, USA [29]	15/15	13/20	80/78	70	MDD; HAM-D≥17	Mild cognitive deficits or mild dementia
Kiosses et al., 2015, USA [30]	37/37	16/14	81	70/78	MDD; MADRS≥17	At least mild cognitive deficit; disability; limited mobility
*Cognitive behavioural therapy*						
Huang et al., 2015, Taiwan [31]	18/19/20	0/0/0	77/76/76	44/58/55	No MDD; GDS-15≥5	N/A
Hyer et al., 2009, USA [32]	13/12	0/0	78/81	16	Depressive disorder; GDS-SF≥5	Long-term care residents
Joling et al., 2011, Netherlands [33]	86/84	21/7	82/81	70/77	No MDD; CES-D>16	Recruited in general practices
Moss et al., 2012,USA [34]	13/13	23/0	79/76	77/77	GDS≥5	N/A
Serfaty et al., 2009, UK [35]	70/67/67	9/13/18	74	79	Depressive disorder; BDI-II≥14	N/A
*Reminiscence therapy/life review*						
Hsu et al., 2009 [36]	21/24	6	78	48/50	GDS-SF≥7	Residents in long-term care facilities
Preschl et al., 2012, Switzerland [37]	21/19	5/16	70	75/56	BDI-II = 10-28	N/A
Serrano et al., 2004, Spain [38]	25/25	20/8	77	83/70	CES-D≥16	Social service clients

BDI-II, Beck Depression Inventory, 2^nd^ edition; CES-D, Center for Epidemiologic Studies Depression Scale; COPD, Chronic Obstructive Pulmonary Disease; GDS-SF, Geriatric Depression Scale Short Form; HAM-D, Hamilton Depression Rating Scale; MADRS, Montgomery-Åsberg Depression Rating Scale; MDD, Major Depressive Disorder; PHQ-2, Patient Healthcare Questionnaire 2

^a^ When the information was reported separately for each treatment group, the data for each group is presented with the intervention groups first.

^b^ Any indicator of frailty, including health condition, cognitive deficits, type of residence, and context from which the study participants were recruited.

**Table 2:** Characteristics of the intervention and comparator in randomized controlled trials of psychological treatment for depression in people aged 65 years and over.

Author, year	Intervention	Duration and intensity	Delivery	Therapists and training	Comparator
Alexopolous et al., 2003 [25]	PST	12 weekly sessions	Individually	6 therapists; 1-day workshop	Supportive therapy
Gellis et al., 2007 [28]	PST	6 1-hour sessions in 8 weeks	Individually in patient’s home	2 social workers	Usual care
Gellis et al., 2010 [26]	PST	6 1-hour sessions in 6 weeks	Individually in patient’s home	Two weeks of training	Usual care + psychoeducation
Gellis et al., 2014 [27]	PST + telemonitoring of physical symptoms	8 weekly 35 min problem solving sessions and daily telemonitoring of physical symptoms over 3 months	Individually over the telephone	3 nurses receiving 6 weeks of supervision	Usual care + psychoeducation
Kiosses et al., 2010 [29]	PST	12 weekly sessions	Individually in patient’s home	3 trained and certified therapists	Supportive therapy
Kiosses et al., 2015 [30]	Problem Adaption Therapy	12 weekly sessions	Individually in the patient’s home	3 clinical psychologists, 4 clinical social workers, 1 clinical doctoral candidate	Supportive therapy
Huang et al., 2015 [31]	CBT	12 weekly sessions lasting 60–80 min	Groups of 3–5	A geriatric nurse; trained in CBT and supervised	1. Exercise;2. Waitlist
Hyer et al., 2009 [32]	CBT (Group, individual and staff therapy, GIST)	13 weekly 75–90 min group sessions, 1–2 individual sessions, and one coach session	Group, individual, and coach sessions	N/A; Staff/peer coach	Treatment as usual
Joling et al., 2011 [33]	CBT (Coping with depression manual)	12 weeks. On average 3 visits and 2 phone calls	Bibliotherapy	Visits by trained home care nurses	Usual care
Moss et al., 2012 [34]	CBT (Overcoming depression one step at a time)	Instructions to complete in 4 weeks. 5–10 min weekly phone contact	Bibliotherapy	N/A	Delayed treatment
Serfaty et al., 2009 [35]	CBT	Up to 12 50-min sessions	Individual sessions	Therapists with at least 5 years practice	1. Talking control; 2. Treatment as usual
Hsu et al., 2009 [36]	Reminiscence therapy	8 weekly sessions	Groups of 8–10	8 geriatric nurse specialists	No treatment
Preschl et al., 2012 [37]	Life review	6 weekly sessions (1–1.5 h)	Each session divided in two parts: face-to-face and computer	2 psychologists with training in CBT. 5 h training in life review and regular supervision	Waitlist
Serrano et al., 2004 [38]	Life review	4 sessions over 3 to 6 weeks	Individual sessions	1 therapist	Usual social assistance

BDI, Beck Depression Inventory; BDI-II, Beck Depression Inventory, 2^nd^ edition; CBT, Cognitive Behaviour Therapy; CES-D, Center for Epidemiologic Studies Depression Scale; GDS, Geriatric Depression Scale; GDS-SF, Geriatric Depression Scale Short Form; HAM-D, Hamilton Depression Rating Scale; LSI-A, Life Satisfaction Index, version A; MADRS, Montgomery-Åsberg Depression Rating Scale; N/A, Not Applicable; NS, Not Significant; PST, Problem-Solving Therapy; QL-Index, Quality of Life Index

### Problem-solving therapy

#### Study characteristics

All six included trials of PST were conducted in the USA: four were relatively small, with fewer than 50 randomized participants, one was based on 74 randomized participants [30] and one on 115 randomized participants [27]. The subjects were predominantly female. The mean age ranged from 74 to 80 years across studies. Three trials included participants with MDD and cognitive deficits [25, 29, 30]. The remaining three trials included participants with various general medical conditions and clinical symptoms of depression, but no confirmed depressive disorder [26–28] (Table 1).

The treatment lasted between 6 and 12 weeks. Weekly sessions were delivered individually, in the patient’s home [26, 28, 29, 30] or by telephone [27]. In one study, the therapy was combined with telemonitoring of physical symptoms [27]. One trial tested a treatment called Problem Adaption Therapy, PATH, which integrated a problem-solving approach and personalized strategies to regulate emotions [30]. The therapists delivering treatment included social workers, nurses, and clinical psychologists. Three trials used supportive therapy as the comparator [25, 29, 30] and in the remainder, the therapy was compared with usual care (Table 2).

#### Results of individual studies

The PATH trial [30] included 74 participants with MDD and at least mild cognitive deficits. Compared with supportive therapy the effect after 12 weeks, measured on the Montgomery-Åsberg Depression Rating Scale, MADRS [39], was *d* = 0.60 (CI 0.13 to 1.06). Participants in PATH had a significantly greater rate of remission (MADRS ≤ 7; 38 vs. 14%, p = .02) and response (≥ 50% reduction on MADRS; 67 vs. 32%, p = .01).

Both the other PTS-trials including participants with a confirmed depressive disorder indicated a significant effect as measured with the Hamilton Depression Rating Scale (HAM-D) [40] after 12 weeks treatment. One of the trials also reported the proportion in remission after the treatment, defined as a HAM-D score below 10 [25]. A significantly greater proportion of subjects receiving PST compared with those receiving supportive therapy achieved remission (75% vs. 22%, p<.01).

All three trials which included participants without a confirmed depressive disorder indicated a significant effect on depressive symptoms, as measured with Beck Depression Inventory (BDI) [41] in two of the trials [26, 28] and the Ham-D and the Patient Health Questionnaire-9 (PHQ-9) [42] in the third trial [27]. The outcome was measured after up to 6 months after baseline in two of the trials [27, 28] and post-treatment only in one trial [26]. The Quality of Life Index (QL-Index) [43] was included as an outcome measure in one of the trials [28] and the results suggested a significant and positive effect (Table 3).

**Table 3:** Outcome of randomized controlled trials of psychological treatment for depression in people aged 65 years and over.

*Intervention*, Author, year	Follow-up period	Depressive symptoms				Other eligible outcomes			
		Measure	Intervention group, mean (SD) at follow-up	Comparator, mean (SD) at follow-up	Hedges *g* (95% CI) favouring the intervention	Outcome (measure)	Intervention group, mean (SD) or % at follow-up	Comparator, mean (SD) or % at follow-up	Hedges *g* (95% CI) favouring the intervention
*Problem-solving therapy*									
Alexopolous et al., 2003 [25]	Post	HAM-D	7.1 (6.3)	13.9 (6.3)	1.05 (0.21; 1.90)	Remission (HAM-D < 10)	75%	22%	Significant difference
Gellis et al., 2007 [28]	6 and 3 months (and post)	BDI	9.69 (7.1)	28.5 (5.4)	2.92 (2.01; 3.84)	QoL (QL-Index)	12.7 (2.0)	8.5 (1.7)	2.21 (1.48; 2.94)
Gellis et al., 2010 [26]	Post	BDI	18.3 (7.7)	25.8 (7.5)	0.96 (0.27; 1.66)	N/A	-	-	-
Gellis et al., 2014 [27]	6 months (and post)	HAM-D	10.4 (7.1)	17.4 (6.3)	1.04 (0.60; 1.47)	Depressive symptoms (PHQ-9)	7.9 (5.3)	14.1 (5.9)	1.10 (0.70; 1.49)
Kiosses et al., 2010 [29]	Post	HAM-D	8.9 (4.1)	12.8 (5.7)	0.75 (0.01; 1.49)	N/A	-	-	-
Kiosses et al., 2015 [30]	Post	MADRS	Reported in a figure	Reported in a figure	Cohen’s *d*, 0.60 (0.13; 1.06)	Remission (MADRS ≤ 7); Response (≥ 50% MADRS reduction)	38%; 67%	14%; 32%	Significant difference
*Cognitive behavioural therapy*									
Huang et al., 2015 [31]	Post, 3 month, 6 month	GDS-15	Reported in a figure	Reported in a figure	Not estimated (data not available)	SF-36	Reported in a figure	Reported in a figure	Not estimated (data not available)
Hyer et al., 2009 [32]	Post	GDS-SF	5.0 (3.5)	10.5 (1.6)	1.93 (0.95; 2.90)	N/A	-	-	-
Joling et al., 2011 [33]	Post	CES-D	16.6 (6.4)	17.3 (6.5)	0.10 (-0.20; 0.40)	Significant improvement (decrease of ≥ 5 CES-D points)	47%	44%	NS
Moss et al., 2012 [34]	Post	HAM-D	5.5 (5.0)	10.8 (5.0)	1.05 (0.22; 1.88)	N/A	-	-	-
Serfaty et al., 2009 [35]	10 months (and 4 months)	BDI-II	18.3 (10.6)	TC: 20.3 (9.0); TAU: 20.8 (10.5)	0.20 (-0.13; 0.54); 0.24 (-0.10; 0.57)	QoL (Euroqol)	0.54 (0.33)	TC: 0.52 (0.32); TAU: 0.52 (0.31)	0.06 (-0.27; 0.40); 0.06 (-0.27; 0.40)
*Reminiscence therapy/life review*									
Hsu et al., 2009 [36]	Post	GDS	7.9 (1.7)	10.7 (2.3)	1.35 (0.69; 2.00)	N/A	-	-	-
Preschl et al., 2012 [37]	Post	BDI-II	10.0 (6.3)	15.1 (7.8)	0.71 (0.03; 1.39)	QoL (LSI-A)	31.6 (3.8)	32.6 (3.4)	-0.27 (-0.93, 0.39)
Serrano et al., 2004 [38]	Post	CES-D	20.5 (7.3)	27.6 (7.5)	0.95 (0.32; 1.59)	QoL (LSI-A)	19.5 (6.5)	14.0 (7.8)	0.74 (0.12; 1.37)

BDI, Beck Depression Inventory; BDI-II, Beck Depression Inventory, 2^nd^ edition; CBT, Cognitive Behaviour Therapy; CES-D, Center for Epidemiologic Studies Depression Scale; GDS, Geriatric Depression Scale; GDS-SF, Geriatric Depression Scale Short Form; HAM-D, Hamilton Depression Rating Scale; LSI-A, Life Satisfaction Index, version A; MADRS, Montgomery-Åsberg Depression Rating Scale; N/A, Not Applicable; NS, Not Significant; PST, Problem-Solving Therapy; QL-Index, Quality of Life Index; QoL, Quality of Life; TAU, Treatment As Usual; TC, Talking Control

#### Synthesis and quality of evidence

Due to the heterogeneity of the populations, the trials including participants with a confirmed depressive disorder were not synthesized with the trials including participants with only confirmed depressive symptoms.

**Fig 2:**
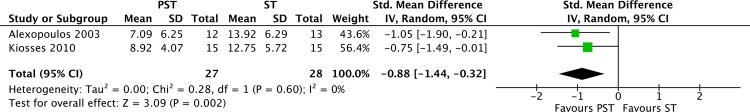
Meta-analysis of randomized controlled trials of problem-solving therapy (PST) vs. supportive therapy (ST) for elderly with a depressive disorder.

A meta-analysis of the two PST-trials including participants with a confirmed depressive disorder suggested a large effect size (Hedges’ *g* = 0.88; 95% CI: 0.32 to 1.44; I^2^ = 0%) on HAM-D in favour of problem-solving therapy (Fig 2). However, the quality of evidence across these studies was rated as very low for all outcomes, due to the risk of bias, imprecision (small sample), and indirectness of the evidence (unclear generalizability from the sample). Because of differences in treatment components, the PATH trial was not synthesized with the two PST trials. Despite the high quality of the PATH study, the quality of evidence was rated as very low (a single trial) (Table 4).

**Table 4:** Quality of evidence supporting psychological treatment of depressive disorders in people aged 65 years and over.

Intervention (I)	Outcome (measure)	Comparator (C)	N/Trials [Reference(s)]	Indicators of frailty	Result	Quality of evidence (GRADE)	Comment
CBT (individual)	Depressive symptoms (BDI-II)	Talking control	137/1 RCT [35]	N/A	I > C	⊕○○○	Single study
CBT (individual)	Quality of Life(Euroqol)	Talking control	137/1 RCT [35]	N/A	No significant difference	⊕○○○	Single study
CBT (individual)	Depressive symptoms (BDI-II)	Usual care	137/1 RCT [35]	N/A	I > C	⊕○○○	Single study
CBT (individual)	Quality of Life (Euroqol)	Usual care	137/1 RCT [35]	N/A	No significant difference	⊕○○○	Single study
CBT (group, individual, staff)	Depressive symptoms (GDS-SF)	Waitlist	25/1 RCT [32]	Long-term care residents	I > C	⊕○○○	Single study
Problem Adaption Therapy	Depressive symptoms (MADRS)	Supportive therapy	74/1 RCT [30]	Cognitive deficits; limited mobility; disability	I > C	⊕○○○	Single study
Problem Adaption Therapy	Remission (MADRS ≤ 7)	Supportive therapy	74/1 RCT [30]	Cognitive deficits; limited mobility; disability	I > C	⊕○○○	Single study
Problem Adaption Therapy	Response (≥ 50% MADRS reduction)	Supportive therapy	74/1 RCT [30]	Cognitive deficits; limited mobility; disability	I > C	⊕○○○	Single study
Problem-solving therapy	Depressive symptoms (HAM-D)	Supportive therapy	55/2 RCT [25, 29]	Mild cognitive deficits	I > C	⊕○○○	-1 risk of bias; -1 indirectness; -1 imprecision
Problem-solving therapy	Remission (HAM-D < 10)	Supportive therapy	25/1 RCT [25]	Mild cognitive deficits	I > C	⊕○○○	Single study

BDI-II, Beck Depression Inventory, 2^nd^ edition; C, Control; CBT, Cognitive Behaviour Therapy; GDS-SF, Geriatric Depression Scale Short Form; HAM-D, Hamilton Depression Rating Scale; I, Intervention; MADRS, Montgomery-Åsberg Depression Rating Scale

**Fig 3:**
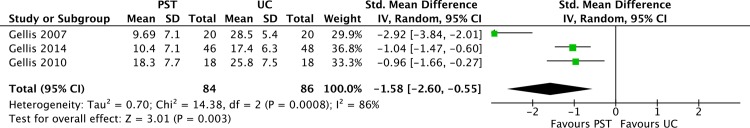
Meta-analysis of randomized controlled trials of problem-solving therapy (PST) vs. usual care (UC) for elderly with depressive symptoms.

A meta-analysis of the three trials including participants with depressive symptoms suggested a large but heterogeneous effect on depressive symptoms (Hedges’ *g* = 1.34; 95% CI: 0.67 to 2.0212; I^2^ = 86%) in favour of problem-solving therapy (Fig 3). Two of the trials included both post-treatment assessments and follow-up assessments 6 month after baseline [27, 28]. The follow-up assessment was included in the meta-analysis, but sensitivity analyses suggested similar results if the post-treatment assessment was used instead. Although all trials suggested a large positive effect of the treatment compared to usual care, one of the trials reported a substantially larger effect than the other two, resulting in substantial heterogeneity. The quality of evidence for effect on depressive symptoms was rated as low across studies due to the risk of bias and indirectness (generalizability from the sample unclear). The true effect may be substantially different from the estimated effect (Table 5).

**Table 5:** Quality of evidence supporting psychological treatment of depressive symptoms in people aged 65 years and over.

Intervention (I)	Outcome (measure)	Comparator (C)	N/Trials [Reference(s)]	Markers of frailty	Result	Quality of evidence (GRADE)	Comment
CBT (bibliotherapy)	Depressive symptoms (CES-D; HAM-D)	Waitlist	196/2 [33, 34]	N/A	Inconsistent	⊕○○○	-1 risk of bias; -1 indirectness; -1 inconsistency
CBT (group-based)	Depressive symptoms (GDS-15)	Waitlist	38/1 [31]	N/A	I > C	⊕○○○	-1 risk of bias; -1 indirectness; -1 imprecision
CBT (group-based)	Quality of Life (SF-36)	Waitlist	38/1 [31]	N/A	No significant difference	⊕○○○	-1 risk of bias; -1 indirectness; -1 imprecision
Problem-solving therapy	Depressive symptoms (BDI; HAM-D)	Usual care	170/3 [26–28]	General medical conditions	I > C	⊕⊕○○	-1 risk of bias; -1 indirectness
Problem-solving therapy	Quality of Life (QL-Index)	Usual care	40/1 [28]	General medical conditions	I > C	⊕○○○	Single study
Reminiscence therapy/life review	Depressive symptoms (BDI-II; CES-D; GDS)	Waitlist	124/3 [36–38]	Varied across studies	I > C	⊕○○○	-1 risk of bias; -1 indirectness; -1 imprecision
Reminiscence therapy/life review	Quality of Life (LSI-A)	Waitlist	79/2 [37, 38]	Varied across studies	Inconsistent	⊕○○○	-1 risk of bias; -1 indirectness; -1 inconsistency

BDI, Beck Depression Inventory; BDI-II, Beck Depression Inventory, 2^nd^ edition; C, Control; CES-D, Center for Epidemiologic Studies Depression Scale; CBT, Cognitive Behaviour Therapy; GDS, Geriatric Depression Scale; HAM-D, Hamilton Depression Rating Scale; I, Intervention; LSI-A, Life Satisfaction Index, version A; QL-Index, Quality of Life Index

The uncertainty surrounding the estimate notwithstanding, based on the fact that several trials with heterogeneous populations and treatment formats indicate a positive effect, the overall judgement is that it is highly probable that PST can have a positive effect on depressive symptoms in frail elderly.

### Cognitive behavioural therapy

#### Study characteristics

The five included CBT-trials were conducted in the USA [32, 34], the Netherlands [33], the UK [35], and Taiwan [31]. The trials were heterogeneous with respect to interventions (mode of delivery, intensity and duration) and the included populations (Tables 1 and 2). Two trials evaluated bibliotherapy with minimal therapist support [33, 34], one evaluated a combination of group, individual, and staff delivered therapy [32], one evaluated group-based CBT [31], and one evaluated individual CBT [35]. In two trials the participants had a confirmed depressive disorder [32, 35]. The level of frailty varied across studies (Table 1).

The two studies of bibliotherapy included predominantly female community dwelling participants with depressive symptoms but no confirmed depressive disorder. One of these studies [34] included 26 participants, randomized to either the self-help manual “Overcoming depression one step at a time” with instructions to complete the steps within 4 weeks, or to a waitlist. The manual is based on the principles for behavioural activation. The other study [33] randomized 170 participants recruited in general practices to the self-help manual “Coping with depression” or usual care for 12 weeks.

The trial evaluating the group, individual, and staff delivered therapy included 25 long-term care residents, predominantly male, with depressive disorder [32]. The participants were randomized to the intervention or usual care groups for 14 weeks. The trial of group-based CBT randomized 57 otherwise healthy participants with depressive symptoms to the therapy, exercise, or waitlist [31]. Finally, the trial of individual CBT included 204 participants with depressive disorder, recruited from primary care [35], randomized to up to 12 sessions of CBT, to a talking control, or to treatment as usual (Table 2).

#### Results of individual studies

A large effect on Ham-D was reported in the bibliotherapy trial using the manual “Overcoming depression one step at a time” [34]. However, the trial using the manual “Coping with depression” [33] did not suggest that the treatment was superior to usual care in terms of the effect on depressive symptoms, measured on the Center for Epidemiologic Studies Depression Scale, CES-D [44]. The group, individual, and staff delivered therapy [32] was reported to be superior to usual care, with a large effect on depressive symptoms, measured on the Geriatric Depression Scale, GDS [45]. In the trial of group-based CBT versus exercise and waitlist [31], depressive symptoms were measured using GDS-15. The results were presented in figures, which did not allow data extraction. However, the presented analyses indicated that the participants receiving the group-based intervention improved more in their depressive symptoms than those on waitlist from pre- to post-treatment, but not to follow-up after 3 and 6 month. Quality of life was measured using the Short Form Health Survey (SF-36) [46] in this trial. The analyses did not suggest a significant difference between the group-treatment and the waitlist on this measure. The difference between the group-treatment and exercise was not analysed. In the trial of individual CBT [35], the results at endpoint and 10 months after baseline suggested that ratings on the Beck Depression Inventory, 2^nd^ edition (BDI-II) [47] were about 2 points lower for the CBT group than for the other groups. This trial also included the EuroQol [48] as a measure of health-related quality of life. No significant difference between the groups was observed for this outcome measure (Table 3).

#### Synthesis and quality of evidence

**Fig 4:**
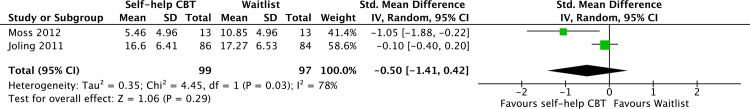
Meta-analysis of randomized controlled trials of self-help cognitive behaviour therapy (CBT) vs. waitlist for elderly with depressive symptoms.

Due to the heterogeneity of the interventions and samples, a meta-analysis of all the studies of CBT was not deemed feasible. A meta-analysis of the two studies of bibliotherapy indicated heterogeneous effects and inconsistent results (Fig 4). Across studies of bibliotherapy for depressive symptoms in people aged 65 or over, the quality of evidence was rated as very low, on the basis of risk of bias, inconsistency, and indirectness (generalizability from the sample unclear) (Table 5).

The quality of evidence was also rated as very low for the group-based and the group, individual, and staff delivered therapies on the basis of risk of bias, imprecision (small sample), and indirectness of the evidence (generalizability from the sample unclear). Despite the high quality of the included study of individual CBT, it is a single trial and therefore the quality of evidence for the efficacy of this treatment was also rated as very low for both outcome measures (Table 4).

### Reminiscence therapy or Life review

#### Study characteristics

The three included trials of reminiscence therapy or life review were conducted in Spain [38], Switzerland [37], and Taiwan [36]. All trials were relatively small, with between 40 and 50 randomized participants. The participants did not have a confirmed depressive disorder in any of these studies. The mean age ranged from 70 to 78 across studies. No trial used an active comparator. The trials were heterogeneous with respect to the interventions, mode of delivery and duration, and the participants’ level of frailty (Tables 1 and 2).

The study from Taiwan evaluated eight group sessions of reminiscence therapy for residents in long-term care facilities. The study from Switzerland evaluated six sessions of life review for elderly: each session was delivered face-to-face, with a computer supplement. Finally, the trial from Spain tested four individual sessions of life review for elderly social service clients.

#### Results of individual studies

All three included studies reported comparably large effect sizes post treatment, albeit on different measures (GDS, BDI-II, and Ham-D). Two of the trials [37, 38] included the Life Satisfaction Index, version A (LSI-A), [49] as a measure of QoL. One trial [38] indicated a significant effect of the intervention on this measure, while the other did not [37] (Table 3).

#### Synthesis and quality of evidence

**Fig 5:**
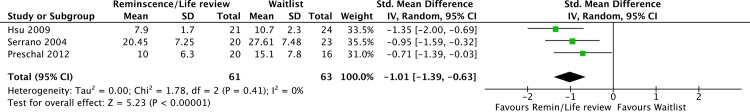
Meta-analysis of randomized controlled trials of reminiscence therapy vs. waitlist for elderly with depressive symptoms.

A meta-analysis of the three included trials suggested that the intervention had a large and homogeneous effect on depressive symptoms (Hedges’ *g* = 1.01; 95% CI: 0.63 to 1.39; I^2^ = 0%; Fig 5). Despite the positive effects suggested by the results, the quality of evidence for all outcomes across studies was rated as very low due to the risk of bias (attrition, no intention to treat analyses), imprecision (small sample), and indirectness (generalizability from the sample unclear) (Table 5).

### Safety

No information about deterioration, adverse events, or any other harmful effects was presented in any of the included trials. No trial indicated that such effects had been monitored. The quality of evidence for safety was therefore graded as very low for all interventions.

### Cost-effectiveness

One RCT from UK comparing CBT, a talking control (TC, in addition to treatment as usual) and treatment as usual (TAU), delivered in a primary care setting was included [16, 35]. The results are for the 10-month follow-up. Complete cost data were available for 198 of 204 patients (mean age 74). Total costs, including intervention and health services costs (mean intervention cost) per patient were estimated in GBP at 1 464 (437), 884 (180), and 1 037 (-) for CBT, TC, and TAU, respectively (currency values for year 2008). Reductions in BDI-II scores were significantly greater in the CBT group than in the other groups: the mean reductions in score were 9.7, 6.0, and 6.2 for CBT, TC, and TAU respectively. There were no significant inter-group differences in health-related quality of life and consequently this was not used in the cost-effectiveness analysis. Cost-effectiveness was estimated at £167 and £120 for CBT compared to TC and TAU, respectively, per point reduction in BDI-II score. The quality of evidence was rated as very low (only one trial).

## Discussion

This systematic review revealed a paucity of studies on the efficacy, safety and cost-effectiveness of psychological treatment of depression for people aged 65 years or over. In accordance with previous reviews, there was support for the efficacy of PST. However, the trials were small and the quality of the evidence across studies was low. Despite the fact that CBT is by far the most frequently studied form of psychological treatment for depression across age groups [50], only five trials were included here. Although the results were promising, the quality of the available evidence was assessed as very low. Also the few available trials on reminiscence therapy/life review showed positive results, although the quality of evidence was very low. Overall, no safety data were reported. Further, no firm conclusions could be drawn from the only eligible cost-effectiveness study [16].

Despite the relatively low number of identified trials, the results clearly suggest that psychological treatment can be a viable alternative for people aged 65 years and over. It should also be noted that estimated effects in trials including younger participants probably are valid also for a substantial proportion of older adults. The age restriction applied to the present review resulted in a different picture compared with that conveyed in more inclusive reviews. There is for instance a striking difference between this review, in which only 14 trials were included, and the recent review by Cuijpers and colleagues, which included 44 trials [9]. However, generalizability across age groups is unclear. Moreover, the most appropriate definition of elderly presumably varies between countries and over time. For instance, the present OECD definition of elderly population, which we used, obviously has less relevance for countries with lower life expectancy.

Generalizability across levels of frailty and types of depression also need to be considered, given that this can vary markedly between service settings (e.g., primary care, psychiatric services, long-term facilities). Deficits related to old age are likely to be more important for treatment outcome than the individuals’ age per se. The participants’ level of frailty was not reported based on established models [13] in any of the included trials. However, several of the PST trials explicitly recruited participants with clear indicators of frailty (e.g., cognitive deficits and cardiovascular disease). Some additional trials recruited participants from settings where age-related deficits are highly prevalent (e.g., long-term care facilities). On the other hand, most of the CBT trials included depressed but otherwise healthy elderly individuals. Cognitive functioning in particular is likely to be a crucial factor in psychological treatment. To our knowledge there is little research on the role of cognitive function as a predictor of outcome in depression treatments for older people. PST is a less complex intervention than CBT, and might therefore be well suited for people with cognitive deficits. Several of the PST trials were explicitly designed for older adults with depression and cognitive deficits. Also generalizability across depressive disorders and severity of depressive symptoms was unclear. The participants in most trials had significant symptoms of depression, but were not formally diagnosed with a depressive disorder. Once again, PST is an exception, as both participants with MDD and subthreshold depressive conditions were included across trials.

There are several additional challenges for future research. First, scant information was forthcoming about the long-term effects of treatment and prevention of relapse. These aspects are of great importance, as it is possible that psychological treatments might help to prevent recurrence even after the treatment has ended [51]. Second, because of the small number of trials and the small samples in most trials it was not possible to generate any meaningful data about moderators of outcome. In addition, no further subgroups could be created given the lack of studies. Given the limited funding available for psychological treatment research, researchers should carefully consider consistency of research designs and outcome measures, in order to facilitate patient-level meta-analyses [52], as these can be used to generate better power for identifying moderators of outcome. This might be particularly important for older patients, given the heterogeneity in symptoms and comorbidity in this age group. A third issue is the absence of information that is vital for proper assessment of the interventions. No information about deterioration, adverse events, or any other harmful effects was presented in any of the trial reports. This is a general shortcoming in psychotherapy research [53]. Future studies need to document and report any negative effects of treatment. Similarly, there was a general lack of information about the use of resources and costs associated with the different treatments – both in terms of implementation and as an outcome of the treatment. Moreover, there is a lack of interpretable cost-effectiveness data for different treatments. Such information is pivotal to justify the implementation of treatment measures and to assist policy makers in judicious decisions about allocation of resources.

### Limitations

Some limitations of our review should be noted. Firstly, we limited the review to RCTs. It is possible, however, that study designs other than RCTs would have given valuable insight. For instance, large cohort studies might provide information about the effects on low frequency outcomes such as suicide and suicidal behaviour. Secondly, we solely relied on information available in the published reports. Some reports did not, for instance, clearly indicate the number of individuals included in the analyses. In such cases we assumed that all randomized participants were included, which might not always be the case. Thirdly, and related, we could not properly assess the risk of publication bias. The low number of included trials – in combination with the heterogeneous interventions, comparators, and populations – made statistical tests of publication bias unreliable. It should be noted, however, that some of the larger trials reported modest effects or no effect, while some of the smaller trials reported remarkably large effect sizes. Thus, we cannot rule out publication bias.

Finally, the assessment of risk of bias and the use of GRADE in the present review might be regarded as overly stringent. Exclusion of trials with a high risk of bias reduced the number of included trials. However, given that the quality of depression trials is likely to influence the effect size estimates [54] we believe that this practice was justified. Further, a major advantage of GRADE is that it provides a framework for guidance through the critical components of the assessment and provides an approach to analysis and communication that encourages transparency and an explicit accounting of the judgements involved [14].

## Conclusions

Despite the limitations and the relatively few studies included, we conclude that PST is a promising treatment for frail elderly people with depressive symptoms and that psychological treatment can be a viable option for this group. However, important questions about efficacy, generalizability, safety and cost-effectiveness still need to be addressed. In the future it will be important to investigate the effects of psychological treatments in the old-old group, formed by complex patients with high frequency of severe comorbid conditions.

## Supporting Information

S1 Appendix Search strategy. (DOCX)

S2 Appendix Excluded publications. (DOCX)

S3 Appendix PRISMA check-list. (DOC)
