# Supplementary material for: Psychological Treatment of Depression in People Aged 65 Years and Over: A Systematic Review of Efficacy, Safety, and Cost-Effectiveness
Source: PLoS One. 2016 Aug 18;11(8):e0160859. doi: 10.1371/journal.pone.0160859 (PMC4990289; doi:10.1371/journal.pone.0160859)
Supplement: S1 Appendix — (DOCX) [file pone.0160859.s001.docx]

S1 Appendix. Search strategy

Efficacy and safety

| Cinahl via EBSCO 5 May 2015 (updated 23 May 2016)  Title: Treatment of late-life depression | | |
| --- | --- | --- |
|  | **Search terms** | **Items found** |
| ***Population: elderly (>65)*** | | |
|  | (MH "Aged") OR (MH "Aged, 80 and Over") OR (MH "Aged, Hospitalized") OR (MH "Frail Elderly") OR (MH "Geriatrics") OR (MH "Aging") | 344,720 |
|  | TI ("older patient" OR "older patients" OR "older adult" OR "older adults" OR "older person" OR "older persons" OR "older women" OR "older men" OR geriatric OR geriatrics OR elderly OR elders OR elder OR senior OR seniors OR 'community dwelling' OR "nursing home" OR "nursing homes" OR "care home" OR "care homes" OR "oldest old" OR frail OR postmenopaus OR postmenopausal OR 'post menopaus' OR 'post menopausal' OR 'late life' OR 'latest life' OR "old age") | 71,283 |
|  | TX “In old age” | 68,751 |
|  | *1 OR 2 OR 3* | *359,395* |
| ***Population: persons with depression*** | | |
|  | MM "Depression" OR (MM "Dysthymic Disorder") OR (MM "Depression, Reactive") OR (MM "Seasonal Affective Disorder") | 24,443 |
|  | TI (depress* OR unipolar OR MDD) | 22,706 |
|  | *5 OR 6* | *29,016* |
| ***Study types: RCT*** | | |
|  | (MH "Randomized Controlled Trials") OR (MH "Random Assignment") | 47,101 |
|  | (TX ((randomized OR randomised) N2 controlled N2 (trial OR study))) NOT (TI systematic review) | 64,738 |
|  | *8 OR 9* | *90,221* |
| ***Combined sets*** | | |
|  | **4 AND 7 AND 10** | **713** |

The search result, usually found at the end of the documentation, forms the list of abstracts

AB = Abstract

AU = Author

DE = Term from the thesaurus

MH = Term from the “Cinahl Headings” thesaurus

MM = Major Concept

TI = Title

TX = All Text. Performs a keyword search of all the  database's searchable fields

ZC = Methodology Index

* = Truncation

“ “ = Citation Marks; searches for an exact phrase

| Cochrane Library via Wiley 5 May 2015 (updated 23 May 2016; CENTRAL)  Title: Treatment of late-life depression | | |
| --- | --- | --- |
|  | **Search terms** | **Items found** |
| ***Population: elderly (>65)*** | | |
|  | "Aged"[Mesh] OR "Aged, 80 and over"[Mesh] OR "Frail Elderly"[Mesh] OR "Geriatrics"[Mesh] | 1034 |
|  | (“older patient*” OR “older adult” OR “older adults” OR “older women” OR “older men” OR geriatric OR geriatrics OR elderly OR elders OR elder OR senior OR seniors OR “community-dwelling” OR “nursing home” Or “nursing homes” OR “care home” OR “care homes” OR “oldest old” OR frail or postmenopaus* OR post-menopaus* OR late-life OR latest-life* OR “old age”):ti | 22023 |
|  | *1 OR 2* | *22485* |
| ***Population: persons with depression*** | | |
|  | "Depressive Disorder, Major"[Mesh] OR "Depression"[Mesh] OR "Depressive Disorder"[Mesh:NoExp] OR Depressive Disorder, Treatment-Resistant[MeSH] OR Dysthymic Disorder[MeSH] OR Seasonal Affective Disorder[MeSH] | 12292 |
|  | (depress* OR unipolar OR MDD):ti | 16536 |
|  | *4 OR 5* | 21443 |
| ***Combined sets*** | | |
|  | **3 AND 6** | **Central/1386** |

The search result, usually found at the end of the documentation, forms the list of abstracts

[AU] = Author

[MAJR] = MeSH Major Topic

[MeSH] = Term from the Medline controlled vocabulary, including terms found below this term in the MeSH hierarchy

[MeSH:NoExp] = Does not include terms found below this term in the MeSH hierarchy

Systematic[SB] = Filter for retrieving systematic reviews

[TI] = Title

[TIAB] = Title or abstract

[TW] = Text Word

* = Truncation

“ “ = Citation Marks; searches for an exact phrase

CDSR = Cochrane Database of Systematic Review

CENTRAL = Cochrane Central Register of Controlled Trials, “trials”

CRM = Method Studies

DARE = Database Abstracts of Reviews of Effects, “other reviews”

EED = Economic Evaluations

HTA = Health Technology Assessments

| EMBASE via Embase.com 5 May 2015 (updated 23 May 2016)  Title: Treatment of late-life depression | | |
| --- | --- | --- |
|  | **Search terms** | **Items found** |
| ***Population: elderly (>65)*** | | |
|  | 'aged'/de OR 'aged hospital patient'/de OR 'frail elderly'/de OR 'very elderly'/de OR 'geriatric patient'/de OR 'elderly care'/exp | 1,503,864 |
|  | "older patient":ti OR "older patients":ti OR "older adult":ti OR "older adults":ti OR "older person":ti OR "older persons":ti OR "older women":ti OR "older men":ti OR geriatric:ti OR geriatrics:ti OR elderly:ti OR elders:ti OR elder:ti OR senior:ti OR seniors:ti OR 'community dwelling':ti OR "nursing home":ti OR "nursing homes":ti OR "care home":ti OR "care homes":ti OR "oldest old":ti OR frail:ti OR postmenopaus:ti OR postmenopausal:ti OR 'post menopaus':ti OR 'post menopausal':ti OR 'late life':ti OR 'latest life':ti OR "old age":ti OR (older NEAR/2 people):ti | 165,104 |
|  | *1 OR 2* | *1545426* |
| ***Population: persons with depression*** | | |
|  | 'depression'/mj OR 'agitated depression'/mj OR 'atypical depression'/mj OR 'depressive psychosis'/mj OR 'dysthymia'/mj OR 'endogenous depression'/mj OR 'involutional depression'/mj OR 'major depression'/mj OR 'masked depression'/mj OR 'melancholia'/mj OR 'mixed anxiety and depression'/mj OR 'mixed depression and dementia'/mj OR 'mourning syndrome'/mj OR 'organic depression'/mj OR 'pseudodementia'/mj OR 'reactive depression'/mj OR 'recurrent brief depression'/mj OR 'seasonal affective disorder'/mj OR 'treatment resistant depression'/mj | 133,323 |
|  | depress*:ti OR unipolar:ti OR MDD:ti | 102,178 |
|  | *4 OR 5* | *157,487* |
| ***Combined sets*** | | |
|  | *(3 AND 6) OR 'late life depression'/de* | 24,562 |
| ***Study types: RCT (modified SIGN search filter)*** | | |
|  | 'randomized controlled trial'/de OR 'randomization'/de OR 'single blind procedure'/de OR 'double blind procedure'/de OR 'crossover procedure'/de OR 'placebo'/de | 710,120 |
|  | (randomi*e*:ti NOT ('meta analysis':ti OR systematic AND review:ti)) OR Random allocation:ab OR Randomly allocated:ab OR Allocated randomly:ab OR (allocated adj2 random):ab OR Single blind*:ab OR Double blind*:ab | 150,556 |
|  | *8 OR 9* | *539,673* |
| ***Combined sets*** | | |
|  | **7 AND 10** | **2,768** |

/de= Term from the EMTREE controlled vocabulary

/exp= Includes terms found below this term in the EMTREE hierarchy

/mj = Major Topic

:ab = Abstract

:au = Author

:ti = Article Title

:ti:ab = Title or abstract

* = Truncation

“ “ = Citation Marks; searches for an exact phrase

| PsycInfo via EBSCO 5 May 2015 (updated 23 May 2016)  Title: Treatment of late-life depression | | |
| --- | --- | --- |
|  | **Search terms** | **Items found** |
| ***Population: elderly (>65)*** | | |
|  | DE "Geriatric Patients" OR DE "Nursing Homes" OR (ZG "aged (65 yrs & older)") or (ZG "very old (85 yrs & older)") OR DE "Geriatrics" OR DE "Gerontology" | 225,489 |
|  | TI ("older patient" OR "older patients" OR "older adult" OR "older adults" OR "older person" OR "older persons" OR "older women" OR "older men" OR geriatric OR geriatrics OR elderly OR elders OR elder OR senior OR seniors OR 'community dwelling' OR "nursing home" OR "nursing homes" OR "care home" OR "care homes" OR "oldest old" OR frail OR postmenopaus OR postmenopausal OR 'post menopaus' OR 'post menopausal' OR 'late life' OR 'latest life' OR "old age") | 54,176 |
|  | TX "In old age" | 12,272 |
|  | *1 OR 2 OR 3* | *239,059* |
| ***Population: persons with depression*** | | |
|  | DE "Major Depression" OR DE "Treatment Resistant Depression" OR DE "Depression (Emotion)" OR (DE "Psychology" AND DE "Symptoms") OR DE "Psychiatric Symptoms" OR DE "Dysthymic Disorder" OR DE "Reactive Depression" OR DE "Recurrent Depression" OR DE "Atypical Depression" OR DE "Pseudodementia" OR DE "Seasonal Affective Disorder" | 119,452 |
|  | TI (depress* OR unipolar OR MDD) | 81,526 |
|  | KW (depression OR "depressive symptoms" OR "Psychiatric Symptoms" OR "psychological symptoms") | 93,660 |
|  | *5 OR 6 OR 7* | *133,501* |
| ***Study types: RCT*** | | |
|  | ((DE "Clinical Trials" OR ZC "treatment outcome/clinical trial") AND (TX random* OR placebo OR TX (singl* n1 blind*) OR TX (doubl* n1 blind*) OR (tripl* n1 blind*) OR (trebl* n1 blind*))) | 20,014 |
|  | TX ((randomized OR randomised) N2 controlled N2 (trial OR study)) | 20,353 |
|  | *9 OR 10* | 32,431 |
| ***Combined sets*** | | |
|  | **4 AND 8 AND 11** | **1,438** |

The search result, usually found at the end of the documentation, forms the list of abstracts

AB = Abstract

AU = Author

DE = Term from the thesaurus

MH = Term from the “Cinahl Headings” thesaurus

MM = Major Concept

TI = Title

TX = All Text. Performs a keyword search of all the  database's searchable fields

ZC = Methodology Index

* = Truncation

“ “ = Citation Marks; searches for an exact phrase

| Pubmed via NLM 5 May 2015 (updated 23 May 2016)  Title: Treatment of late-life depression | | |
| --- | --- | --- |
|  | **Search terms** | **Items found** |
| ***Population: elderly (>65)*** | | |
|  | "Aged"[Mesh] OR "Aged, 80 and over"[Mesh] OR "Frail Elderly"[Mesh] OR "Geriatrics"[Mesh] | 2320161 |
|  | (older patient*[TI] OR older adult[TI] OR older adults[TI] OR older women[TI] OR older men[TI] OR geriatric[TI] OR geriatrics[TI] OR elderly[TI] OR elders[TI] OR elder[TI] OR senior[TI] OR seniors[TI] OR community-dwelling[TIab] OR nursing home[TI] Or nursing homes[TI] OR care home[TI] OR care homes[TI] OR oldest old[TI] OR frail[TI] or postmenopaus*[TI] OR post-menopaus*[ti] OR late-life[ti] OR latest-life*[ti] OR old age[ti]) NOT (medline[SB] OR oldmedline[SB]) | 14224 |
|  | *1 OR 2* | *2334385* |
| ***Population: persons with depression*** | | |
|  | "Depressive Disorder, Major"[Mesh] OR "Depression"[Mesh] OR "Depressive Disorder"[Mesh:NoExp] OR Depressive Disorder, Treatment-Resistant[MeSH] OR Dysthymic Disorder[MeSH] OR Seasonal Affective Disorder[MeSH] | 147275 |
|  | (depress*[ti] OR unipolar[ti] OR MDD[ti]) NOT (medline[SB] OR oldmedline[SB]) | 9492 |
|  | *4 OR 5* | *156767* |
| ***Study types: RCT, PubMed Clinical Queries Filters specific/narrow (modified with added term)*** | | |
|  | (randomized controlled trial[Publication Type] OR ((randomized[Title/Abstract] OR randomised[tiab]) AND controlled[Title/Abstract] AND (trial[Title/Abstract] OR study[tiab])) | 420533 |
| ***Combined sets*** | | |
|  | **3 AND 6 AND 7** | **3796** |

The search result, usually found at the end of the documentation, forms the list of abstracts

[MeSH] = Term from the Medline controlled vocabulary, including terms found below this term in the MeSH hierarchy

[MeSH:NoExp] = Does not include terms found below this term in the MeSH hierarchy

[MAJR] = MeSH Major Topic

[TIAB] = Title or abstract

[TI] = Title

[AU] = Author

[TW] = Text Word

Systematic[SB] = Filter for retrieving systematic reviews

* = Truncation

“ “ = Citation Marks; searches for an exact phrase

| Scopus 5 May 2015 (updated 23 May 2016)  Title: Treatment of late-life depression | | |
| --- | --- | --- |
|  | **Search terms** | **Items found** |
| ***Population: elderly (>65)*** | | |
|  | TITLE("older patient" OR "older patients" OR "older adult" OR "older adults" OR "older person" OR "older persons" OR "older women" OR "older men" OR geriatric OR geriatrics OR elderly OR elders OR elder OR senior OR seniors OR "community dwelling" OR "nursing home" OR "nursing homes" OR "care home" OR "care homes" OR "oldest old" OR frail OR postmenopaus OR postmenopausal OR "post menopaus" OR "post menopausal" OR "late life" OR "latest life" OR "old age") | 246,690 |
| ***Population: persons with depression*** | | |
|  | TITLE (depress* OR unipolar OR MDD) | 136,458 |
| ***Study types: RCT*** | | |
|  | TITLE-ABS-KEY(((randomized OR randomised) W/2 controlled W/2 (trial OR study))) | 477,571 |
| ***Combined sets*** | | |
|  | **1 AND 2 AND 3** | **619** |

The search result, usually found at the end of the documentation, forms the list of abstracts

AB = Abstract

AU = Author

DE = Term from the thesaurus

MH = Term from the “Cinahl Headings” thesaurus

MM = Major Concept

TI = Title

TX = All Text. Performs a keyword search of all the  database's searchable fields

ZC = Methodology Index

* = Truncation

“ “ = Citation Marks; searches for an exact phrase

Health Economics

| Cinahl via EBSCO 5 May 2015 (updated 23 May 2016)  Title: Treatment of late-life depression –Health Economics | | |
| --- | --- | --- |
|  | **Search terms** | **Items found** |
| ***Population: elderly (>65)*** | | |
|  | (MH "Aged") OR (MH "Aged, 80 and Over") OR (MH "Aged, Hospitalized") OR (MH "Frail Elderly") OR (MH "Geriatrics") OR (MH "Aging") | 344,720 |
|  | TI ("older patient" OR "older patients" OR "older adult" OR "older adults" OR "older person" OR "older persons" OR "older women" OR "older men" OR geriatric OR geriatrics OR elderly OR elders OR elder OR senior OR seniors OR 'community dwelling' OR "nursing home" OR "nursing homes" OR "care home" OR "care homes" OR "oldest old" OR frail OR postmenopaus OR postmenopausal OR 'post menopaus' OR 'post menopausal' OR 'late life' OR 'latest life' OR "old age") | 89,970 |
|  | TX "In old age" | 68,751 |
|  | *1 OR 2 OR 3* | *359,402* |
| ***Population: persons with depression*** | | |
|  | MM "Depression" OR (MM "Dysthymic Disorder") OR (MM "Depression, Reactive") OR (MM "Seasonal Affective Disorder") | 24,443 |
|  | TI (depress* OR unipolar OR MDD) | 22,707 |
|  | *5 OR 6* | *29,017* |
| ***Health Economics*** | | |
|  | (MH "Economics") OR (MH "Costs and Cost Analysis+") OR (MH "Economic Aspects of Illness") OR (MH "Economic Value of Life") OR (MH "Economics, Dental") OR (MH "Economics, Pharmaceutical") OR (MH "Fees and Charges+") OR (MH "Quality-Adjusted Life Years") | 70,791 |
|  | (TI "cost utility" OR "cost/utility" OR "cost/benefit" OR "cost benefit" OR "cost effectiveness" OR "cost of illness" OR "cost analysis" OR "cost consequence" OR "cost consequences" OR "cost saving" Or "cost savings" OR "cost breakdown" OR "cost lowering" OR "cost loweings" OR "cost estimate" OR "cost estimates" OR "cost variable" OR "cost variables" OR "cost allocation" OR "cost control" OR "cost per unit" OR "value for money" OR pharmacoeconomic* OR "icer" OR economic evaluation* OR "economic analyses" OR "economic study" OR "economic studies" OR qaly* OR "quality adjusted life" OR "disability adjusted life" OR daly* OR qald* OR qale* OR qtime* OR "hye" OR "hyes" OR "healthy year equivalent" OR "healthy year equivalents" OR "healthy years equivalent" OR "healthy years equivalents" OR "quality of well being" OR "qwb" OR "euroqol" OR "eq5d" OR "eq 5d" OR "health utilities index" OR "hui" OR "hui2" OR "hui3" OR "rosser" OR "sf 6" OR "sf6" OR "sf6D" OR "sf 6D" OR "short form 6D" OR "time trade off" OR "time tradeoff" OR "standard gamble" OR "willingness to pay" OR "willingness to accept" OR "willing to pay") OR (AB "cost utility" OR "cost/utility" OR "cost/benefit" OR "cost benefit" OR "cost effectiveness" OR "cost of illness" OR "cost analysis" OR "cost consequence" OR "cost consequences" OR "cost saving" Or "cost savings" OR "cost breakdown" OR "cost lowering" OR "cost loweings" OR "cost estimate" OR "cost estimates" OR "cost variable" OR "cost variables" OR "cost allocation" OR "cost control" OR "cost per unit" OR "value for money" OR pharmacoeconomic* OR "icer" OR economic evaluation* OR "economic analyses" OR "economic study" OR "economic studies" OR qaly* OR "quality adjusted life" OR "disability adjusted life" OR daly* OR qald* OR qale* OR qtime* OR "hye" OR "hyes" OR "healthy year equivalent" OR "healthy year equivalents" OR "healthy years equivalent" OR "healthy years equivalents" OR "quality of well being" OR "qwb" OR "euroqol" OR "eq5d" OR "eq 5d" OR "health utilities index" OR "hui" OR "hui2" OR "hui3" OR "rosser" OR "sf 6" OR "sf6" OR "sf6D" OR "sf 6D" OR "short form 6D" OR "time trade off" OR "time tradeoff" OR "standard gamble" OR "willingness to pay" OR "willingness to accept" OR "willing to pay") | 41,804 |
|  | TI (economic* OR cost OR costs OR costing OR costly) | 28,745 |
|  | *8 OR 9 OR 10* | 87,617 |
| ***Combined sets*** | | |
|  | **4 AND 7 AND 11** | **181** |

The search result, usually found at the end of the documentation, forms the list of abstracts

AB = Abstract

AU = Author

DE = Term from the thesaurus

MH = Term from the “Cinahl Headings” thesaurus

MM = Major Concept

TI = Title

TX = All Text. Performs a keyword search of all the  database's searchable fields

ZC = Methodology Index

* = Truncation

“ “ = Citation Marks; searches for an exact phrase

| Cochrane Library via Wiley 5 May 2015 (updated 23 May 2016; Economic Evaluations, EED)  Title: Treatment of late-life depression –Health Economics | | |
| --- | --- | --- |
|  | **Search terms** | **Items found** |
| ***Population: elderly (>65)*** | | |
|  | "Aged"[Mesh] OR "Aged, 80 and over"[Mesh] OR "Frail Elderly"[Mesh] OR "Geriatrics"[Mesh] | 1034 |
|  | (“older patient*” OR “older adult” OR “older adults” OR “older women” OR “older men” OR geriatric OR geriatrics OR elderly OR elders OR elder OR senior OR seniors OR “community-dwelling” OR “nursing home” Or “nursing homes” OR “care home” OR “care homes” OR “oldest old” OR frail or postmenopaus* OR post-menopaus* OR late-life OR latest-life* OR “old age”):ti | 22023 |
|  | *1 OR 2* | *22485* |
| ***Population: persons with depression*** | | |
|  | "Depressive Disorder, Major"[Mesh] OR "Depression"[Mesh] OR "Depressive Disorder"[Mesh:NoExp] OR Depressive Disorder, Treatment-Resistant[MeSH] OR Dysthymic Disorder[MeSH] OR Seasonal Affective Disorder[MeSH] | 12292 |
|  | (depress* OR unipolar OR MDD):ti | 16536 |
|  | *4 OR 5* | 21443 |
| ***Combined sets*** | | |
|  | **3 AND 6** | **EED/17** |

The search result, usually found at the end of the documentation, forms the list of abstracts

[AU] = Author

[MAJR] = MeSH Major Topic

[MeSH] = Term from the Medline controlled vocabulary, including terms found below this term in the MeSH hierarchy

[MeSH:NoExp] = Does not include terms found below this term in the MeSH hierarchy

Systematic[SB] = Filter for retrieving systematic reviews

[TI] = Title

[TIAB] = Title or abstract

[TW] = Text Word

* = Truncation

“ “ = Citation Marks; searches for an exact phrase

CDSR = Cochrane Database of Systematic Review

CENTRAL = Cochrane Central Register of Controlled Trials, “trials”

CRM = Method Studies

DARE = Database Abstracts of Reviews of Effects, “other reviews”

EED = Economic Evaluations

HTA = Health Technology Assessments

| EMBASE via Embase.com, 5 May 2015 (updated 23 May 2016)  Title: Treatment of late-life depression –Health Economics | | |
| --- | --- | --- |
|  | **Search terms** | **Items found** |
| ***Population: elderly (>65)*** | | |
|  | 'aged'/de OR 'aged hospital patient'/de OR 'frail elderly'/de OR 'very elderly'/de OR 'geriatric patient'/de OR 'elderly care'/exp | 1,505,539 |
|  | "older patient":ti OR "older patients":ti OR "older adult":ti OR "older adults":ti OR "older person":ti OR "older persons":ti OR "older women":ti OR "older men":ti OR geriatric:ti OR geriatrics:ti OR elderly:ti OR elders:ti OR elder:ti OR senior:ti OR seniors:ti OR 'community dwelling':ti OR "nursing home":ti OR "nursing homes":ti OR "care home":ti OR "care homes":ti OR "oldest old":ti OR frail:ti OR postmenopaus:ti OR postmenopausal:ti OR 'post menopaus':ti OR 'post menopausal':ti OR 'late life':ti OR 'latest life':ti OR "old age":ti OR (older NEAR/2 people):ti | 165,549 |
|  | *1 OR 2* | *1,547,314* |
| ***Population: persons with depression*** | | |
|  | 'depression'/mj OR 'agitated depression'/mj OR 'atypical depression'/mj OR 'depressive psychosis'/mj OR 'dysthymia'/mj OR 'endogenous depression'/mj OR 'involutional depression'/mj OR 'major depression'/mj OR 'masked depression'/mj OR 'melancholia'/mj OR 'mixed anxiety and depression'/mj OR 'mixed depression and dementia'/mj OR 'mourning syndrome'/mj OR 'organic depression'/mj OR 'pseudodementia'/mj OR 'reactive depression'/mj OR 'recurrent brief depression'/mj OR 'seasonal affective disorder'/mj OR 'treatment resistant depression'/mj | 133,491 |
|  | depress*:ti OR unipolar:ti OR MDD:ti | 102,425 |
|  | *4 OR 5* | 157,789 |
| ***Combined sets*** | | |
|  | (3 AND 6) OR 'late life depression'/de | 24,589 |
| ***Health Economics*** | | |
|  | ('health economics'/de/mj OR 'economic evaluation'/exp/mj OR 'health-care-cost'/exp/mj OR 'pharmacoeconomics'/exp/mj OR 'economic aspect'/exp/mj OR 'financial management'/exp/mj OR ((economic near/2 (evaluation* OR analys* OR stud*)) OR (value* near/2 (money or monetary)) OR pharmacoeconomic* OR (pharmaco next/1economic*) OR "icer" OR (cost NEAR/2 (util* OR benefit* OR effectiveness OR illness OR analys* OR consequence* OR saving* OR breakdown* OR low* OR high* OR estimate* OR variable* OR allocation* OR control* OR unit))):ti,ab OR (economic* OR cost*):ti OR 'quality adjusted life year'/de/mj OR utility:ti,ab OR utilities:ti,ab OR qaly*:ti,ab OR "quality adjusted life":ti,ab OR "disability adjusted life":ti,ab OR daly*:ti,ab OR qald*:ti,ab OR qale*:ti,ab OR qtime*:ti,ab OR "hye":ti,ab OR "hyes":ti,ab OR (('healthy year' OR 'healthy years' OR 'health year' OR 'health years') NEXT/2 equivalent*):ti,ab OR "quality of well being":ti,ab OR "qwb":ti,ab OR "euroqol":ti,ab OR "eq5d":ti,ab OR "eq 5d":ti,ab OR "health utilities index":ti,ab OR "hui":ti,ab OR "hui2":ti,ab OR "hui3":ti,ab OR "rosser":ti,ab OR "sf 6":ti,ab OR "sf6":ti,ab OR "sf6D":ti,ab OR "sf 6D":ti,ab OR "short form 6D":ti,ab OR "time trade off":ti,ab OR "time tradeoff":ti,ab OR "standard gamble":ti,ab OR ((willing or willingness) near/2 (pay or accept)):ti,ab) | 405,244 |
| ***Combined sets*** | | |
|  | **7 AND 8** | **782** |

/de= Term from the EMTREE controlled vocabulary

/exp= Includes terms found below this term in the EMTREE hierarchy

/mj = Major Topic

:ab = Abstract

:au = Author

:ti = Article Title

:ti:ab = Title or abstract

* = Truncation

“ “ = Citation Marks; searches for an exact phrase

| PsycInfo via EBSCO 5 May 2015 (updated 23 May 2016)  Title: Treatment of late-life depression –Health Economics | | |
| --- | --- | --- |
|  | **Search terms** | **Items found** |
| ***Population: elderly (>65)*** | | |
|  | DE "Geriatric Patients" OR DE "Nursing Homes" OR (ZG "aged (65 yrs & older)") or (ZG "very old (85 yrs & older)") OR DE "Geriatrics" OR DE "Gerontology" | 225,489 |
|  | TI ("older patient" OR "older patients" OR "older adult" OR "older adults" OR "older person" OR "older persons" OR "older women" OR "older men" OR geriatric OR geriatrics OR elderly OR elders OR elder OR senior OR seniors OR 'community dwelling' OR "nursing home" OR "nursing homes" OR "care home" OR "care homes" OR "oldest old" OR frail OR postmenopaus OR postmenopausal OR 'post menopaus' OR 'post menopausal' OR 'late life' OR 'latest life' OR "old age") | 54,176 |
|  | TX "In old age" | 12,272 |
|  | *1 OR 2 OR 3* | *239,059* |
| ***Population: persons with depression*** | | |
|  | DE "Major Depression" OR DE "Treatment Resistant Depression" OR DE "Depression (Emotion)" OR (DE "Psychology" AND DE "Symptoms") OR DE "Psychiatric Symptoms" OR DE "Dysthymic Disorder" OR DE "Reactive Depression" OR DE "Recurrent Depression" OR DE "Atypical Depression" OR DE "Pseudodementia" OR DE "Seasonal Affective Disorder" | 119,452 |
|  | TI (depress* OR unipolar OR MDD) | 81,526 |
|  | KW (depression OR "depressive symptoms" OR "Psychiatric Symptoms" OR "psychological symptoms") | 93,660 |
|  | *5 OR 6 OR 7* | *133,501* |
| ***Economic studies*** | | |
|  | DE "Costs and Cost Analysis" OR DE "Budgets" OR DE "Health Care Costs" OR (TI cost OR Costs) OR (TX Cost effective* OR cost benefit*) | 68,338 |
| ***Combined sets*** | | |
|  | **4 AND 8 AND 9** | **419** |

The search result, usually found at the end of the documentation, forms the list of abstracts

AB = Abstract

AU = Author

DE = Term from the thesaurus

MH = Term from the “Cinahl Headings” thesaurus

MM = Major Concept

TI = Title

TX = All Text. Performs a keyword search of all the  database's searchable fields

ZC = Methodology Index

* = Truncation

“ “ = Citation Marks; searches for an exact phrase

| Pubmed via NLM 5 May 2015 (updated 23 May 2016)  Title: Treatment of late-life depression –Health Economics | | |
| --- | --- | --- |
|  | **Search terms** | **Items found** |
| ***Population: elderly (>65)*** | | |
|  | "Aged"[Mesh] OR "Aged, 80 and over"[Mesh] OR "Frail Elderly"[Mesh] OR "Geriatrics"[Mesh] | 2320970 |
|  | (older patient*[TI] OR older adult[TI] OR older adults[TI] OR older women[TI] OR older men[TI] OR geriatric[TI] OR geriatrics[TI] OR elderly[TI] OR elders[TI] OR elder[TI] OR senior[TI] OR seniors[TI] OR community-dwelling[TIab] OR nursing home[TI] Or nursing homes[TI] OR care home[TI] OR care homes[TI] OR oldest old[TI] OR frail[TI] or postmenopaus*[TI] OR post-menopaus*[ti] OR late-life[ti] OR latest-life*[ti] OR old age[ti]) NOT (medline[SB] OR oldmedline[SB]) | 14279 |
|  | *1 OR 2* | *2335249* |
| ***Population: persons with depression*** | | |
|  | "Depressive Disorder, Major"[Mesh] OR "Depression"[Mesh] OR "Depressive Disorder"[Mesh:NoExp] OR Depressive Disorder, Treatment-Resistant[MeSH] OR Dysthymic Disorder[MeSH] OR Seasonal Affective Disorder[MeSH] | 147336 |
|  | (depress*[ti] OR unipolar[ti] OR MDD[ti]) NOT (medline[SB] OR oldmedline[SB]) | 9514 |
|  | *4 OR 5* | *156850* |
| ***Economic studies*** | | |
|  | (cost*[Title/Abstract] OR "costs and cost analysis"[MeSH:noexp] OR cost benefit analys*[Title/Abstract] OR cost-benefit analysis[MeSH Term] OR health care costs[MeSH:noexp]) | 413579 |
| ***Combined sets*** | | |
|  | **3 AND 6 AND 7** | **884** |

The search result, usually found at the end of the documentation, forms the list of abstracts

[MeSH] = Term from the Medline controlled vocabulary, including terms found below this term in the MeSH hierarchy

[MeSH:NoExp] = Does not include terms found below this term in the MeSH hierarchy

[MAJR] = MeSH Major Topic

[TIAB] = Title or abstract

[TI] = Title

[AU] = Author

[TW] = Text Word

Systematic[SB] = Filter for retrieving systematic reviews

* = Truncation

“ “ = Citation Marks; searches for an exact phrase

| Scopus 5 May 2015 (updated 23 May 2016)  Title: Treatment of late-life depression –Health Economics | | |
| --- | --- | --- |
|  | **Search terms** | **Items found** |
| ***Population: elderly (>65)*** | | |
|  | TITLE("older patient" OR "older patients" OR "older adult" OR "older adults" OR "older person" OR "older persons" OR "older women" OR "older men" OR geriatric OR geriatrics OR elderly OR elders OR elder OR senior OR seniors OR "community dwelling" OR "nursing home" OR "nursing homes" OR "care home" OR "care homes" OR "oldest old" OR frail OR postmenopaus OR postmenopausal OR "post menopaus" OR "post menopausal" OR "late life" OR "latest life" OR "old age") | 246,793 |
| ***Population: persons with depression*** | | |
|  | TITLE (depress* OR unipolar OR MDD) | 136,524 |
| ***Economic studies*** | | |
|  | (TI cost OR Costs) OR (TX Cost effective* OR cost benefit* OR cost analysis) | 64,671 |
| ***Combined sets*** | | |
|  | **1 AND 2 AND 3** | **35** |

The search result, usually found at the end of the documentation, forms the list of abstracts
