# Supplementary material for: Psychological Treatment of Depression in People Aged 65 Years and Over: A Systematic Review of Efficacy, Safety, and Cost-Effectiveness
Source: PLoS One. 2016 Aug 18;11(8):e0160859. doi: 10.1371/journal.pone.0160859 (PMC4990289; doi:10.1371/journal.pone.0160859)
Supplement: S2 Appendix — (DOCX) [file pone.0160859.s002.docx]

**S2 Appendix: Excluded publications with reasons**

Table of Contents

Excluded publications – efficacy and safety 1

Population 1

Intervention 21

Outcome 22

Study design 22

Type of publication 24

Language 27

Duplicates 28

Excluded publications – cost-effectiveness 28

Population 28

Outcome 34

Type of publication 35

Excluded publications – efficacy and safety

Population

1. Cognitive therapy for depression. Bandolier. 2005;12(5):1-2

2. Alexopoulos GS, Raue PJ, McCulloch C, Kanellopoulos D, Seirup JK, Sirey JA, et al. Clinical Case Management versus Case Management with Problem-Solving Therapy in Low-Income, Disabled Elders with Major Depression: A Randomized Clinical Trial. American Journal of Geriatric Psychiatry. 2016;24(1):50-9.

3. Al-Otaiba Z, Epstein EE, McCrady B, Cook S. Age-based differences in treatment outcome among alcohol-dependent women. Psychology of addictive behaviors : journal of the Society of Psychologists in Addictive Behaviors. 2012;26(3):423-31.

4. Anderson-Hanley C, Meshberg SR, Marsh MA. The effects of a control-enhancing intervention for nursing home residents: cognition and locus of control as moderators. Palliat Support Care. 2003;1:111-20.

5. Ando M, Morita T, Akechi T, Okamoto T, Japanese Task Force for S, Care. Efficacy of short-term life-review interviews on the spiritual well-being of terminally ill cancer patients. J Pain Symptom Manage. 2010;39:993-1002.

6. Apostolo JL, Cardoso DF, Rosa AI, Paul C. The effect of cognitive stimulation on nursing home elders: a randomized controlled trial. Journal of nursing scholarship : an official publication of Sigma Theta Tau International Honor Society of Nursing / Sigma Theta Tau. 2014;46(3):157-66.

7. Apóstolo JLA, Cardoso DFB, Rosa AI, Paúl C. The Effect of Cognitive Stimulation on Nursing Home Elders: A Randomized Controlled Trial. Journal of Nursing Scholarship. 2014;46(3):157-66.

8. Appels A, van E, Bar F, van der P, Erdman RA, Assman M, et al. Effects of a behavioural intervention on quality of life and related variables in angioplasty patients: results of the EXhaustion Intervention Trial. J Psychosom Res. 2006;61:1-7; discussion 9.

9. Arean PA, Perri MG, Nezu AM, Schein RL, Christopher F, Joseph TX. Comparative effectiveness of social problem-solving therapy and reminiscence therapy as treatments for depression in older adults. J Consult Clin Psychol. 1993;61(6):1003-10.

10. Arean PA, Raue P, Mackin RS, Kanellopoulos D, McCulloch C, Alexopoulos GS. Problem-solving therapy and supportive therapy in older adults with major depression and executive dysfunction. Am J Psychiatry. 2010;167:1391-8.

11. Axford J, Heron C, Ross F, Victor CR. Management of knee osteoarthritis in primary care: pain and depression are the major obstacles. J Psychosom Res. 2008;64:461-7.

12. Bailey EM, Stevens AB, LaRocca MA, Scogin F. A Randomized Controlled Trial of a Therapeutic Intervention for Nursing Home Residents With Dementia and Depressive Symptoms. J Appl Gerontol. 2016.

13. Baker AL, Kavanagh DJ, Kay-Lambkin FJ, Hunt SA, Lewin TJ, Carr VJ, et al. Randomized controlled trial of cognitive-behavioural therapy for coexisting depression and alcohol problems: short-term outcome. Addiction. 2010;105:87-99.

14. Bakker Ton JEM, Duivenvoorden Hugo J, van der L, Jacqueline, Olde R, Marcel GM, et al. Integrative psychotherapeutic nursing home program to reduce multiple psychiatric symptoms of cognitively impaired symptoms of cognitively impaired patients and caregiver burden: Randomized controlled trial. The American Journal of Geriatric Psychiatry. 2011;19:507-20.

15. Beckie TM, Beckstead JW, Schocken DD, Evans ME, Fletcher GF. The effects of a tailored cardiac rehabilitation program on depressive symptoms in women: A randomized clinical trial. Int J Nurs Stud. 2011;48:3-12.

16. Bedi N, Chilvers C, Churchill R, Dewey M, Duggan C, Fielding K, et al. Assessing effectiveness of treatment of depression in primary care. Partially randomised preference trial. Br J Psychiatry. 2000;177:312-8.

17. Berman RL, Iris MA, Bode R, Drengenberg C. The effectiveness of an online mind-body intervention for older adults with chronic pain. J Pain. 2009;10:68-79.

18. Bichescu D, Neuner F, Schauer M, Elbert T. Narrative exposure therapy for political imprisonment-related chronic posttraumatic stress disorder and depression. Behav Res Ther. 2007;45:2212-20.

19. Black JL, Allison TG, Williams DE, Rummans TA, Gau GT. Effect of intervention for psychological distress on rehospitalization rates in cardiac rehabilitation patients. Psychosomatics. 1998;39:134-43.

20. Bolton P, Bass J, Neugebauer R, Verdeli H, Clougherty KF, Wickramaratne P, et al. Group Interpersonal Psychotherapy for Depression in Rural Uganda: A Randomized Controlled Trial. Journal of the American Medical Association. 2003;289:3117-24.

21. Bond GE, Burr RL, Wolf FM, Feldt K. The effects of a web-based intervention on psychosocial well-being among adults aged 60 and older with diabetes: a randomized trial. Diabetes Educ. 2010;36:446-56.

22. Bosmans JE, Schreuders B, van Marwijk HW, Smit JH, van Oppen P, van Tulder MW. Cost-effectiveness of problem-solving treatment in comparison with usual care for primary care patients with mental health problems: a randomized trial. BMC Fam Pract. 2012;13:98.

23. Breitbart W, Poppito S, Rosenfeld B, Vickers AJ, Li Y, Abbey J, et al. Pilot randomized controlled trial of individual meaning-centered psychotherapy for patients with advanced cancer. J Clin Oncol. 2012;30:1304-9.

24. Brodaty H, Draper BM, Millar J, Low LF, Lie D, Sharah S, et al. Randomized controlled trial of different models of care for nursing home residents with dementia complicated by depression or psychosis. J Clin Psychiatry. 2003;64:63-72.

25. Broderick JE, Junghaenel DU, Schneider S, Bruckenthal P, Keefe FJ. Treatment expectation for pain coping skills training: relationship to osteoarthritis patients' baseline psychosocial characteristics. Clin J Pain. 2011;27:315-22.

26. Brody BL, Roch-Levecq AC, Kaplan RM, Moutier CY, Brown SI. Age-related macular degeneration: self-management and reduction of depressive symptoms in a randomized, controlled study. J Am Geriatr Soc. 2006;54(10):1557-62.

27. Buckwalter KC, Gerdner L, Kohout F, Hall GR, Kelly A, Richards B, et al. A nursing intervention to decrease depression in family caregivers of persons with dementia. Arch Psychiatr Nurs. 1999;13:80-8.

28. Burns A, Banerjee S, Morris J, Woodward Y, Baldwin R, Proctor R, et al. Treatment and prevention of depression after surgery for hip fracture in older people: randomized, controlled trials. J Am Geriatr Soc. 2007;55:75-80.

29. Burns DS. The effect of the bonny method of guided imagery and music on the mood and life quality of cancer patients. J Music Ther. 2001;38:51-65.

30. Catalan J, Gath DH, Anastasiades P, Bond SA, Day A, Hall L. Evaluation of a brief psychological treatment for emotional disorders in primary care. Psychol Med. 1991;21:1013-8.

31. Cavanagh K, Seccombe N, Lidbetter N. The implementation of computerized cognitive behavioural therapies in a service user-led, third sector self help clinic. Behav Cogn Psychother. 2011;39:427-42.

32. Cavanagh K, Shapiro DA, Van Den B, Swain S, Barkham M, Proudfoot J. The effectiveness of computerized cognitive behavioural therapy in routine care. Br J Clin Psychol. 2006;45:499-514.

33. Cernin PA, Lichtenberg PA. Behavioral treatment for depressed mood: a pleasant events intervention for seniors residing in assisted living. Clinical Gerontologist. 2009;32(3):324-31.

34. Chan MF, Chan EA, Mok E. Effects of music on depression and sleep quality in elderly people: A randomised controlled trial. Complement Ther Med. 2010;18:150-9.

35. Chan MF, Ng SE, Tien A, Man Ho RC, Thayala J. A randomised controlled study to explore the effect of life story review on depression in older Chinese in Singapore. Health Soc Care Community. 2013;21(5):545-53.

36. Chan MF, Wong ZY, Onishi H, Thayala NV. Effects of music on depression in older people: a randomised controlled trial. J Clin Nurs. 2012;21:776-83.

37. Chang BL. Cognitive-behavioral intervention for homebound caregivers of persons with dementia. Nurs Res. 1999;48(3):173-82.

38. Chew-Graham CA, Lovell K, Roberts C, Baldwin R, Morley M, Burns A, et al. A randomised controlled trial to test the feasibility of a collaborative care model for the management of depression in older people. The British journal of general practice : the journal of the Royal College of General Practitioners. 2007;57:364-70.

39. Chiang KJ, Chu H, Chang HJ, Chung MH, Chen CH, Chiou HY, et al. The effects of reminiscence therapy on psychological well-being, depression, and loneliness among the institutionalized aged. Int J Geriatr Psychiatry. 2010;25:380-8.

40. Chiesa A, Castagner V, Andrisano C, Serretti A, Mandelli L, Porcelli S, et al. Mindfulness-based cognitive therapy vs. Psycho-education for patients with major depression who did not achieve remission following antidepressant treatment. Psychiatry Research. 2015;226:474-83.

41. Chippendale T, Bear-Lehman J. Effect of life review writing on depressive symptoms in older adults: a randomized controlled trial. Am J Occup Ther. 2012;66:438-46.

42. Choi I, Zou J, Titov N, Dear BF, Li S, Johnston L, et al. Culturally attuned internet treatment for depression amongst Chinese Australians: A randomised controlled trial. J Affect Disord. 2012;136(3):459-68.

43. Choi NG, Marti CN, Bruce ML, Hegel MT. Depression in homebound older adults: problem-solving therapy and personal and social resourcefulness. Behav Ther. 2013;44(3):489-500.

44. Choi NG, Marti CN, Bruce ML, Hegel MT, Wilson NL, Kunik ME. Six-month postintervention depression and disability outcomes of in-home telehealth problem-solving therapy for depressed, low-income homebound older adults. Depress Anxiety. 2014;31(8):653-61.

45. Choy JCP, Lou VWQ. Effectiveness of the Modified Instrumental Reminiscence Intervention on Psychological Well-Being Among Community-Dwelling Chinese Older Adults: A Randomized Controlled Trial. American Journal of Geriatric Psychiatry. 2016;24(1):60-9.

46. Chu H, Yang CY, Lin Y, Ou KL, Lee TY, O'Brien AP, et al. The Impact of Group Music Therapy on Depression and Cognition in Elderly Persons With Dementia: A Randomized Controlled Study. Biol Res Nurs. 2013.

47. Chung LJ, Tsai PS, Liu BY, Chou KR, Lin WH, Shyu YK, et al. Home-based deep breathing for depression in patients with coronary heart disease: a randomised controlled trial. Int J Nurs Stud. 2010;47:1346-53.

48. Constantino RE, Sekula LK, Rubinstein EN. Group intervention for widowed survivors of suicide. Suicide Life Threat Behav. 2001;31:428-41.

49. Coon DW, Thompson L, Steffen A, Sorocco K, Gallagher-Thompson D. Anger and depression management: psychoeducational skill training interventions for women caregivers of a relative with dementia. Gerontologist. 2003;43(5):678-89.

50. Crisp D, Griffiths K, Mackinnon A, Bennett K, Christensen H. An online intervention for reducing depressive symptoms: secondary benefits for self-esteem, empowerment and quality of life. Psychiatry Res. 2014;216(1):60-6.

51. Damush TM, Wu J, Bair MJ, Sutherland JM, Kroenke K. Self-management practices among primary care patients with musculoskeletal pain and depression. Journal of behavioral medicine. 2008;31:301-7.

52. Dao TK, Youssef NA, Armsworth M, Wear E, Papathopoulos KN, Gopaldas R. Randomized controlled trial of brief cognitive behavioral intervention for depression and anxiety symptoms preoperatively in patients undergoing coronary artery bypass graft surgery. J Thorac Cardiovasc Surg. 2011;142:e109-15.

53. Davis MC. Life review therapy as an intervention to manage depression and enhance life satisfaction in individuals with right hemisphere cerebral vascular accidents 1997.

54. Davison BJ, Degner LF. Empowerment of men newly diagnosed with prostate cancer. Cancer Nurs. 1997;20:187-96.

55. de G, L E, Hollon SD, Huibers MJ. Predicting outcome in computerized cognitive behavioral therapy for depression in primary care: A randomized trial. J Consult Clin Psychol. 2010;78:184-9.

56. de G, L E, Huibers MJ, Riper H, Gerhards SA, Arntz A. Use and acceptability of unsupported online computerized cognitive behavioral therapy for depression and associations with clinical outcome. J Affect Disord. 2009;116:227-31.

57. DeBerry S. The effects of mediation-relaxation on anxiety and depression in a geriatric population. Psychotherapy. 1982;19:512-21.

58. Dechamps A, Alban R, Jen J, Decamps A, Traissac T, Dehail P. Individualized Cognition-Action intervention to prevent behavioral disturbances and functional decline in institutionalized older adults: a randomized pilot trial. Int J Geriatr Psychiatry. 2010;25:850-60.

59. Dobkin RD, Menza M, Allen LA, Tiu J, Friedman J, Bienfait KL, et al. Telephone-based cognitive-behavioral therapy for depression in Parkinson disease. J Geriatr Psychiatry Neurol. 2011;24:206-14.

60. Dobkin RD, Troster AI, Rubino JT, Allen LA, Gara MA, Mark MH, et al. Neuropsychological outcomes after psychosocial intervention for depression in Parkinson's disease. J Neuropsychiatry Clin Neurosci. 2014;26(1):57-63.

61. Doering LV, Chen B, Cross Bodan R, Magsarili MC, Nyamathi A, Irwin MR. Early cognitive behavioral therapy for depression after cardiac surgery. J Cardiovasc Nurs. 2013;28(4):370-9.

62. Dolbeault S, Cayrou S, Bredart A, Viala AL, Desclaux B, Saltel P, et al. The effectiveness of a psycho-educational group after early-stage breast cancer treatment: results of a randomized French study. Psychooncology. 2009;18:647-56.

63. Dombrovski AY, Lenze EJ, Dew MA, Mulsant BH, Pollock BG, Houck PR, et al. Maintenance treatment for old-age depression preserves health-related quality of life: a randomized, controlled trial of paroxetine and interpersonal psychotherapy. J Am Geriatr Soc. 2007;55:1325-32.

64. Donnan P, Hutchinson A, Paxton R, Grant B, Firth M. Self-help materials for anxiety: a randomized controlled trial in general practice. The British journal of general practice : the journal of the Royal College of General Practitioners. 1990;40:498-501.

65. Doorenbos A, Given B, Given C, Verbitsky N. Physical functioning: effect of behavioral intervention for symptoms among individuals with cancer. Nurs Res. 2006;55:161-71.

66. Downe-Wamboldt BL, Butler LJ, Melanson PM, Coulter LA, Singleton JF, Keefe JM, et al. The effects and expense of augmenting usual cancer clinic care with telephone problem-solving counseling. Cancer Nurs. 2007;30:441-53.

67. Edelman S, Bell DR, Kidman AD. A group cognitive behaviour therapy programme with metastatic breast cancer patients. Psychooncology. 1999;8:295-305.

68. Eller LS, Kirksey KM, Nicholas PK, Corless IB, Holzemer WL, Wantland DJ, et al. A randomized controlled trial of an HIV/AIDS Symptom Management Manual for depressive symptoms. AIDS Care - Psychological and Socio-Medical Aspects of AIDS/HIV. 2013;25:391-9.

69. Erdley SD, Gellis ZD, Bogner HA, Kass DS, Green JA, Perkins RM. Problem-solving therapy to improve depression scores among older hemodialysis patients: a pilot randomized trial. Clin Nephrol. 2014;82(1):26-33.

70. Ersek M, Turner JA, Cain KC, Kemp CA. Results of a randomized controlled trial to examine the efficacy of a chronic pain self-management group for older adults [ISRCTN11899548]. Pain. 2008;138:29-40.

71. Escobar JI, Gara MA, Diaz-Martinez AM, Interian A, Warman M, Allen LA, et al. Effectiveness of a time-limited cognitive behavior therapy type intervention among primary care patients with medically unexplained symptoms. Ann Fam Med. 2007;5:328-35.

71. Favrod J, Rexhaj S, Bardy S, Ferrari P, Hayoz C, Moritz S, et al. Sustained antipsychotic effect of metacognitive training in psychosis: A randomized-controlled study. European Psychiatry. 2014;29:275-81.

72. Fledderus M, Bohlmeijer ET, Pieterse ME, Schreurs KMG. Acceptance and commitment therapy as guided self-help for psychological distress and positive mental health: A randomized controlled trial. Psychol Med. 2012;42(3):485-95.

73. Floyd M, Rohen N, Shackelford JA, Hubbard KL, Parnell MB, Scogin F, et al. Two-year follow-up of bibliotherapy and individual cognitive therapy for depressed older adults. Behav Modif. 2006:281-94.

74. Floyd M, Scogin F, McKendree-Smith NL, Floyd DL, Rokke PD. Cognitive therapy for depression: A comparison of individual psychotherapy and bibliotherapy for depressed older adults. Behav Modif. 2004; (2):297-318.

75. Foley E, Baillie A, Huxter M, Price M, Sinclair E. Mindfulness-based cognitive therapy for individuals whose lives have been affected by cancer: a randomized controlled trial. J Consult Clin Psychol. 2010;78:72-9.

76. Freedland KE, Skala JA, Carney RM, Rubin EH, Lustman PJ, Davila-Roman VG, et al. Treatment of depression after coronary artery bypass surgery: a randomized controlled trial. Arch Gen Psychiatry. 2009;66:387-96.

77. Fry PS. Structured and unstructured reminiscence training and depression among the elderly. Clinical Gerontologist. 1983:15-37.

78. Furze G, Dumville JC, Miles JN, Irvine K, Thompson DR, Lewin RJ. "Prehabilitation" prior to CABG surgery improves physical functioning and depression. Int J Cardiol. 2009;132:51-8.

79. Galfin JM, Watkins ER, Harlow T. A brief guided self-help intervention for psychological distress in palliative care patients: a randomised controlled trial. Palliative medicine. 2012;26(3):197-205.

80. Gallagher DE, Thompson LW. Effectiveness of psychotherapy for both endogenous and nonendogenous depression in older adult outpatients. Journals of Gerontology. 1983:707-12.

81. Gallagher-Thompson D, Hanley-Peterson P, Thompson LW. Maintenance of gains versus relapse following brief psychotherapy for depression. J Consult Clin Psychol. 1990;58:371-4.

82. Gallagher-Thompson D, Steffen AM. Comparative effects of cognitive-behavioral and brief psychodynamic psychotherapies for depressed family caregivers. J Consult Clin Psychol. 1994;62(3):543-9.

83. Gallegos AM, Hoerger M, Talbot NL, Moynihan JA, Duberstein PR. Emotional benefits of mindfulness-based stress reduction in older adults: the moderating roles of age and depressive symptom severity. Aging Ment Health. 2013;17(7):823-9.

84. Gander F, Proyer RT, Ruch W, Wyss T. Strength-based positive interventions: Further evidence for their potential in enhancing well-being and alleviating depression. Journal of Happiness Studies. 2013;14(4):1241-59.

85. Gandy M, Sharpe L, Nicholson Perry K, Thayer Z, Miller L, Boserio J, et al. Cognitive behaviour therapy to improve mood in people with epilepsy: a randomised controlled trial. Cogn Behav Ther. 2014;43(2):153-66.

86. Gensichen J, Petersen JJ, Karroum T, Rauck S, Ludman E, Konig J, et al. Positive impact of a family practice-based depression case management on patient's self-management. Gen Hosp Psychiatry. 2011;33:23-8.

87. Giaquinto S, Cacciato A, Minasi S, Sostero E, Amanda S. Effects of music-based therapy on distress following knee arthroplasty. Br J Nurs. 2006;15:576-9.

88. Gitlin LN. Mediators of the impact of a home-based intervention (beat the blues) on depressive symptoms among older African Americans. Psychology and aging. 2014;29(3):601-11.

89. Glueckauf RL, Davis WS, Willis F, Sharma D, Gustafson DJ, Hayes J, et al. Telephone-based, cognitive-behavioral therapy for African American dementia caregivers with depression: initial findings. Rehabil Psychol. 2012;57:124-39.

90. Goerling U, Foerg A, Sander S, Schramm N, Schlag PM. The impact of short-term psycho-oncological interventions on the psychological outcome of cancer patients of a surgical-oncology department - a randomised controlled study. Eur J Cancer. 2011;47:2009-14.

91. Griffiths KM, Mackinnon AJ, Crisp DA, Christensen H, Bennett K, Farrer L. The Effectiveness of an Online Support Group for Members of the Community with Depression: A Randomised Controlled Trial. PLoS One. 2012;7.

92. Guetin S, Ginies P, Siou DK, Picot MC, Pommie C, Guldner E, et al. The effects of music intervention in the management of chronic pain: a single-blind, randomized, controlled trial. Clin J Pain. 2012;28:329-37.

93. Guetin S, Portet F, Picot MC, Pommie C, Messaoudi M, Djabelkir L, et al. Effect of music therapy on anxiety and depression in patients with Alzheimer's type dementia: randomised, controlled study. Dement Geriatr Cogn Disord. 2009;28:36-46.

94. Gustavson KA, Alexopoulos GS, Niu GC, McCulloch C, Meade T, Areán PA. Problem-Solving Therapy Reduces Suicidal Ideation In Depressed Older Adults with Executive Dysfunction. American Journal of Geriatric Psychiatry. 2016;24(1):11-7.

95. Haight BK. The therapeutic role of a structured life review process in homebound elderly subjects. J Gerontol. 1988:P40-4.

96. Haight BK, Michel Y, Hendrix S. Life review: preventing despair in newly relocated nursing home residents short- and long-term effects. Int J Aging Hum Dev. 1998;47:119-42.

97. Haley WE, Bergman EJ, Roth DL, McVie T, Gaugler JE, Mittelman MS. Long-term effects of bereavement and caregiver intervention on dementia caregiver depressive symptoms. Gerontologist. 2008;48:732-40.

98. Hanser SB, Thompson LW. Effects of a music therapy strategy on depressed older adults. J Gerontol. 1994;49:P265-9.

99. Haringsma R, Engels GI, Spinhoven P. Predictors of response to the 'Coping with Depression' course for older adults. A field study. Aging Ment Health. 2006;10:424-34.

100. Heckman TG, Sikkema KJ, Hansen N, Kochman A, Heh V, Neufeld S, et al. A randomized clinical trial of a coping improvement group intervention for HIV-infected older adults. Journal of behavioral medicine. 2011;34:102-11.

101. Hirani SP, Beynon M, Cartwright M, Rixon L, Doll H, Henderson C, et al. The effect of telecare on the quality of life and psychological well-being of elderly recipients of social care over a 12-month period: the Whole Systems Demonstrator cluster randomised trial. Age Ageing. 2014;43(3):334-41.

102. Hoffman CJ, Ersser SJ, Hopkinson JB, Nicholls PG, Harrington JE, Thomas PW. Effectiveness of mindfulness-based stress reduction in mood, breast- and endocrine-related quality of life, and well-being in stage 0 to III breast cancer: a randomized, controlled trial. J Clin Oncol. 2012;30:1335-42.

103. Hollinghurst S, Carroll FE, Abel A, Campbell J, Garland A, Jerrom B, et al. Cost-effectiveness of cognitive-behavioural therapy as an adjunct to pharmacotherapy for treatment-resistant depression in primary care: Economic evaluation of the CoBalT Trial. British Journal of Psychiatry. 2014;204(1):69-76.

104. Hsiao FH, Jow GM, Kuo WH, Chang KJ, Liu YF, Ho RT, et al. The effects of psychotherapy on psychological well-being and diurnal cortisol patterns in breast cancer survivors. Psychother Psychosom. 2012;81:173-82.

105. Hsieh CJ, Chang C, Su SF, Hsiao YL, Shih YW, Han WH, et al. Reminiscence group therapy on depression and apathy in nursing home residents with mild-to-moderate dementia. Journal of Experimental and Clinical Medicine. 2010:72-8.

106. Hu Z, Hu Y, Lu Q. Impact of early rehabilitation therapy on post stroke depression. Chinese Journal of Clinical Rehabilitation. 2003;7:849.

107. Hussain RA, Lawrence PS. Social reinforcement of activity and problem-solving training in the treatment of depressed institutionalized elderly patients. Cognitive Therapy and Research. 1981;5:57-69.

108. Hynninen MJ, Bjerke N, Pallesen S, Bakke PS, Nordhus IH. A randomized controlled trial of cognitive behavioral therapy for anxiety and depression in COPD. Respir Med. 2010;104:986-94.

109. Jarrett RB, Minhajuddin A, Borman PD, Dunlap L, Segal ZV, Kidner CL, et al. Cognitive reactivity, dysfunctional attitudes, and depressive relapse and recurrence in cognitive therapy responders. Behav Res Ther. 2012;50(5):280-6.

110. Jarvik LF, Mintz J, Steuer J, Gerner R. Treating geriatric depression: A 26-week interim analysis. J Am Geriatr Soc. 1982:713-7.

104. Jerant A, Moore M, Lorig K, Franks P. Perceived control moderated the self-efficacy-enhancing effects of a chronic illness self-management intervention. Chronic Illn. 2008;4:173-82.

111. Johns SA, Kroenke K, Theobald DE, Wu J, Tu W. Telecare management of pain and depression in patients with cancer: patient satisfaction and predictors of use. J Ambul Care Manage. 2011;34:126-39.

112. Jonkers CC, Lamers F, Bosma H, Metsemakers JF, van E, J T. The effectiveness of a minimal psychological intervention on self-management beliefs and behaviors in depressed chronically ill elderly persons: a randomized trial. Int Psychogeriatr. 2012;24:288-97.

113 Jordan J, Carter JD, McIntosh VVW, Fernando K, Frampton CMA, Porter RJ, et al. Metacognitive therapy versus cognitive behavioural therapy for depression: a randomized pilot study. Australian & New Zealand Journal of Psychiatry. 2014;48(10):932-43.

114. Julião M, Oliveira F, Nunes B, Vaz Carneiro A, Barbosa A. Efficacy of Dignity Therapy on Depression and Anxiety in Portuguese Terminally Ill Patients: A Phase II Randomized Controlled Trial. Journal of Palliative Medicine. 2014;17(6):688-95.

115. Juliao M, Oliveira F, Nunes B, Vaz Carneiro A, Barbosa A. Efficacy of dignity therapy on depression and anxiety in Portuguese terminally ill patients: a phase II randomized controlled trial. Journal of palliative medicine. 2014;17(6):688-95.

116. Karimi H, Dolatshahee B, Momeni K, Khodabakhshi A, Rezaei M, Kamrani AA. Effectiveness of integrative and instrumental reminiscence therapies on depression symptoms reduction in institutionalized older adults: An empirical study. Aging Ment Health. 2010:881-7.

117. Katon W, Rutter C, Ludman EJ, Von K, Lin E, Simon G, et al. A randomized trial of relapse prevention of depression in primary care. Arch Gen Psychiatry. 2001;58:241-7.

118. Kay-Lambkin FJ, Baker AL, Kelly B, Lewin TJ. Clinician-assisted computerised versus therapist-delivered treatment for depressive and addictive disorders: A randomised controlled trial. Medical Journal of Australia. 2011;195(3 SUPPL.):S44-S50.

119. Kessler D, Lewis G, Kaur S, Wiles N, King M, Weich S, et al. Therapist-delivered internet psychotherapy for depression in primary care: a randomised controlled trial. The Lancet. 2009;374:628-34.

120. King M, Davidson O, Taylor F, Haines A, Sharp D, Turner R. Effectiveness of teaching general practitioners skills in brief cognitive behaviour therapy to treat patients with depression: randomised controlled trial. BMJ (Clinical research ed). 2002;324:947-50.

121. Kitsumban V, Thapinta D, Sirindharo PB, Anders RL. Effect of cognitive mindfulness practice program on depression among elderly Thai women. Thai Journal of Nursing Research. 2009;13(2):95-107.

122. Klausner EJ, Clarkin JF, Spielman L, Pupo C, Abrams R, Alexopoulos GS. Late-life depression and functional disability: the role of goal-focused group psychotherapy. Int J Geriatr Psychiatry. 1998;13:707-16.

123. Klein DN, Leon AC, Li C, D'Zurilla TJ, Black SR, Vivian D, et al. Social problem solving and depressive symptoms over time: a randomized clinical trial of cognitive-behavioral analysis system of psychotherapy, brief supportive psychotherapy, and pharmacotherapy. J Consult Clin Psychol. 2011;79:342-52.

124. Klemm P. Effects of online support group format (moderated vs peer-led) on depressive symptoms and extent of participation in women with breast cancer. Comput Inform Nurs. 2012;30:9-18.

125. Ko HJ, Youn CH. Effects of laughter therapy on depression, cognition and sleep among the community-dwelling elderly. Geriatr Gerontol Int. 2011;11:267-74.

126. Koertge J, Janszky I, Sundin O, Blom M, Georgiades A, Laszlo KD, et al. Effects of a stress management program on vital exhaustion and depression in women with coronary heart disease: a randomized controlled intervention study. J Intern Med. 2008;263:281-93.

127. Kohn CS, Petrucci RJ, Baessler C, Soto DM, Movsowitz C. The effect of psychological intervention on patients' long-term adjustment to the ICD: a prospective study. Pacing Clin Electrophysiol. 2000;23:450-6.

128. Kolanowski AM, Litaker M, Buettner L. Efficacy of theory-based activities for behavioral symptoms of dementia. Nurs Res. 2005;54:219-28.

129. Kong Y, Dong WL, Liu CF. Fluoxetine for poststroke depression: A randomized placebo controlled clinical trial. Neural Regeneration Research. 2007;2:162-5.

130. Konnert C, Dobson K, Stelmach L. The prevention of depression in nursing home residents: a randomized clinical trial of cognitive-behavioral therapy. Aging Ment Health. 2009;13:288-99.

131. Krishnamurthy MN, Telles S. Assessing depression following two ancient Indian interventions: effects of yoga and ayurveda on older adults in a residential home. J Gerontol Nurs. 2007;33(2):17-23.

132. Kroenke K, Theobald D, Wu J, Norton K, Morrison G, Carpenter J, et al. Effect of telecare management on pain and depression in patients with cancer: a randomized trial. JAMA. 2010;304:163-71.

133. Kunik ME, Braun U, Stanley MA, Wristers K, Molinari V, Stoebner D, et al. One session cognitive behavioural therapy for elderly patients with chronic obstructive pulmonary disease. Psychol Med. 2001;31:717-23.

134. Kurtz ME, Kurtz JC, Given CW, Given B. A randomized, controlled trial of a patient/caregiver symptom control intervention: effects on depressive symptomatology of caregivers of cancer patients. J Pain Symptom Manage. 2005;30:112-22.

135. Lai Claudia KY, Chi I, Kayser-Jones J. A randomized controlled trial of a specific reminiscence approach to promote the well-being of nursing home residents with dementia. International Psychogeriatrics. 2004;16:33-49.

136. Lamers F, Jonkers CC, Bosma H, Chavannes NH, Knottnerus JA, van E, et al. Improving quality of life in depressed COPD patients: effectiveness of a minimal psychological intervention. COPD. 2010;7:315-22.

137. Lamers F, Jonkers CC, Bosma H, Kempen GI, Meijer JA, Penninx BW, et al. A minimal psychological intervention in chronically ill elderly patients with depression: a randomized trial. Psychother Psychosom. 2010;79:217-26.

138. Lamers F, Jonkers CC, Bosma H, Knottnerus JA, van E, J T. Treating depression in diabetes patients: does a nurse-administered minimal psychological intervention affect diabetes-specific quality of life and glycaemic control? A randomized controlled trial. J Adv Nurs. 2011;67:788-99.

139. Landreville P, Bissonnette L. Effects of cognitive bibliotherapy for depressed older adults with a disability. Clinical Gerontologist. 1997;17:35-55.

140. Lang AJ. Brief intervention for co-occurring anxiety and depression in primary care: a pilot study. Int J Psychiatry Med. 2003;33(2):141-54.

141. Latorre JM, Serrano JP, Ricarte J, Bonete B, Ros L, Sitges E. Life review based on remembering specific positive events in active aging. J Aging Health. 2015;27(1):140-57.

142. Lavretsky H, Epel ES, Siddarth P, Nazarian N, Cyr NS, Khalsa DS, et al. A pilot study of yogic meditation for family dementia caregivers with depressive symptoms: effects on mental health, cognition, and telomerase activity. Int J Geriatr Psychiatry. 2013;28:57-65.

143. Lenze EJ, Dew MA, Mazumdar S, Begley AE, Cornes C, Miller MD, et al. Combined pharmacotherapy and psychotherapy as maintenance treatment for late-life depression: effects on social adjustment. Am J Psychiatry. 2002;159(3):466-8.

144. Levesque DA, Van M, D F, Schneider RJ, Bauer MR, Goldberg DN, et al. Randomized trial of a computer-tailored intervention for patients with depression. Am J Health Promot. 2011;26:77-89.

145. Little P, Dorward M, Warner G, Moore M, Stephens K, Senior J, et al. Randomised controlled trial of effect of leaflets to empower patients in consultations in primary care. BMJ (Clinical research ed). 2004;328:441.

146. Lopez J, Crespo M. Analysis of the efficacy of a psychotherapeutic program to improve the emotional status of caregivers of elderly dependent relatives. Aging Ment Health. 2008;12(4):451-61.

147. Lovejoy T. Telephone-delivered motivational interviewing targeting sexual risk behavior reduces depression, anxiety, and stress in HIV-positive older adults. Annals of Behavioral Medicine. 2012:416-21.

148. Ludman Evette J, Peterson D, Katon Wayne J, Von K, Michael, Ciechanowski P, et al. Improving Confidence for Self Care in Patients with Depression and Chronic Illnesses. Behavioral Medicine. 2013;39:1-6.

149. Lynch TR, Morse JQ, Mendelson T, Robins CJ. Dialectical behavior therapy for depressed older adults: a randomized pilot study. Am J Geriatr Psychiatry. 2003;11(1):33-45.

150. Manber R, Arnow B, Blasey C, Vivian D, McCullough JP, Blalock JA, et al. Patient's therapeutic skill acquisition and response to psychotherapy, alone or in combination with medication. Psychol Med. 2003;33:693-702.

151. Mantovani G, Astara G, Lampis B, Bianchi A, Curreli L, Orru W, et al. Impact of psychosocial intervention on the quality of life of elderly cancer patients. Psychooncology. 1996;5:127-35.

152. Markle-Reid M, Weir R, Browne G, Roberts J, Gafni A, Henderson S. Health promotion for frail older home care clients. J Adv Nurs. 2006;54:381-95.

153. Martensson J, Stromberg A, Dahlstrom U, Karlsson JE, Fridlund B. Patients with heart failure in primary health care: effects of a nurse-led intervention on health-related quality of life and depression. Eur J Heart Fail. 2005;7:393-403.

154. Mastel-Smith BA, McFarlane J, Sierpina M, Malecha A, Haile B. Geropsychiatry. Improving depressive symptoms in community-dwelling older adults. J Gerontol Nurs. 2007;33:13-9.

155. Mastel-Smith BA, McFarlane J, Sierpina M, Malecha A, Haile B. Improving depressive symptoms in community-dwelling older adults: a psychosocial intervention using life review and writing. J Gerontol Nurs. 2007;33:13-9.

156. Mavandadi S, Benson A, DiFilippo S, Streim JE, Oslin D. A telephone-based program to provide symptom monitoring alone vs symptom monitoring plus care management for late-life depression and anxiety a randomized clinical trial. JAMA Psychiatry. 2015; 72(12):1211-8.

157. Maynard CK. Comparison of effectiveness of group interventions for depression in women. Arch Psychiatr Nurs. 1993;7(5):277-83.

158. McDowell JE, McClean S, FitzGibbon F, Tate S. A randomised clinical trial of the effectiveness of home-based health care with telemonitoring in patients with COPD. Journal of Telemedicine and Telecare. 2015;21(2):80-7.

159. McGeoch GR, Willsman KJ, Dowson CA, Town GI, Frampton CM, McCartin FJ, et al. Self-management plans in the primary care of patients with chronic obstructive pulmonary disease. Respirology. 2006;11:611-8.

160. McLaughlin TJ, Aupont O, Bambauer KZ, Stone P, Mullan MG, Colagiovanni J, et al. Improving psychologic adjustment to chronic illness in cardiac patients. The role of depression and anxiety. J Gen Intern Med. 2005;20:1084-90.

161. Meeks S, Haitsma K, Schoenbachler B, Looney SW. BE-ACTIV for depression in nursing homes: primary outcomes of a randomized clinical trial. The journals of gerontology Series B, Psychological sciences and social sciences. 2015; 70(1):13-23.

162. Meeks S, Young CM, Looney SW. Activity participation and affect among nursing home residents: Support for a behavioural model of depression. Aging Ment Health. 2007; (6):751-60.

163. Merrill Kari A, Tolbert Valerie E, Wade Wendy A. Effectiveness of cognitive therapy for depression in a community mental health center: A benchmarking study. Journal of Consulting and Clinical Psychology. 2003;71:404-9.

164. Meyer B, Bierbrodt J, Schroder J, Berger T, Beevers CG, Weiss M, et al. Effects of an Internet intervention (Deprexis) on severe depression symptoms: Randomized controlled trial. Internet Interventions. 2015;2(1):48-59.

165. Mittelman MS, Roth DL, Coon DW, Haley WE. Sustained benefit of supportive intervention for depressive symptoms in caregivers of patients with Alzheimer's disease. Am J Psychiatry. 2004;161:850-6.

166. Mohammadi Ali Z, Shahabi T, Panah Fereshteh M. An evaluation of the effect of group music therapy on stress, anxiety, and depression levels in nursing home residents. Canadian Journal of Music Therapy. 2011;17:55-68.

167. Mohr DC, Goodkin DE, Islar J, Hauser SL, Genain CP. Treatment of depression is associated with suppression of nonspecific and antigen-specific TH1 responses in multiple sclerosis. Archives of Neurology. 2001;58:1081-6.

168. Moleiro C, Beutler LE. Clinically significant change in psychotherapy for depressive disorders. J Affect Disord. 2009;115(1-2):220-4.

169. Moon Fai C, P. Leong KS, Boon Ling H, Koottappal Mathew B, Banu A. L. Khan S, Sagayamary Lourdusamy S, et al. Reducing depression among community-dwelling older adults using life-story review: A pilot study. Geriatric nursing. 2014;35(2):105-10.

170. Moorey S, Cort E, Kapari M, Monroe B, Hansford P, Mannix K, et al. A cluster randomized controlled trial of cognitive behaviour therapy for common mental disorders in patients with advanced cancer. Psychol Med. 2009;39(5):713-23.

171. Morgan AJ, Jorm AF, Mackinnon AJ. Email-based promotion of self-help for subthreshold depression: Mood Memos randomised controlled trial. Br J Psychiatry. 2012;200:412-8.

172. Morgan MAJ, Coates MJ, Dunbar JA, Reddy P, Schlicht K, Fuller J. The TrueBlue model of collaborative care using practice nurses as case managers for depression alongside diabetes or heart disease: A randomised trial. BMJ Open. 2013;3.

173. Moss AS, Reibel DK, Greeson JM, Thapar A, Bubb R, Salmon J, et al. An adapted mindfulness-based stress reduction program for elders in a continuing care retirement community: quantitative and qualitative results from a pilot randomized controlled trial. J Appl Gerontol. 2015;34(4):518-38.

174. Mossey JM, Knott KA, Higgins M, Talerico K. Effectiveness of a psychosocial intervention, interpersonal counseling, for subdysthymic depression in medically ill elderly. The journals of gerontology Series A, Biological sciences and medical sciences. 1996;51:M172-8.

175. Moynihan C, Bliss JM, Davidson J, Burchell L, Horwich A. Evaluation of adjuvant psychological therapy in patients with testicular cancer: randomised controlled trial. BMJ (Clinical research ed). 1998;316:429-35.

176. Mozley CG, Schneider J, Cordingley L, Molineux M, Duggan S, Hart C, et al. The care home activity project: does introducing an occupational therapy programme reduce depression in care homes? Aging Ment Health. 2007;11(1):99-107.

177. Mundt JC, DeBrota DJ, Greist JH. Anchoring perceptions of clinical change on accurate recollection of the past: Memory enhanced retrospective evaluation of treatment (MERET®). Psychiatry. 2007;4(3):39-45.

178. Murphy SM, Edwards RT, Williams N, Raisanen L, Moore G, Linck P, et al. An evaluation of the effectiveness and cost effectiveness of the National Exercise Referral Scheme in Wales, UK: a randomised controlled trial of a public health policy initiative. J Epidemiol Community Health. 2012;66:745-53.

179. Nance DC. Pains, joys, and secrets: nurse-led group therapy for older adults with depression. Issues Ment Health Nurs. 2012;33:89-95.

180. Newby JM, Mewton L, Williams AD, Andrews G. Effectiveness of transdiagnostic Internet cognitive behavioural treatment for mixed anxiety and depression in primary care. Journal of Affective Disorders. 2014;165:45-52.

181. Ng SE, Tien A, Thayala JNV, Ho RCM, Chan MF. The effect of life story review on depression of older community-dwelling Chinese adults in Singapore: A preliminary result. Int J Geriatr Psychiatry. 2013;28:328-30.

182. Norris CM, Patterson L, Galbraith D, Hegadoren KM. All you have to do is call; a pilot study to improve the outcomes of patients with coronary artery disease. Applied Nursing Research. 2009;22(2):133-7.

183. Okumiya K, Morita Y, Nishinaga M, Osaki Y, Doi Y, Ishine M, et al. Effects of group work programs on community-dwelling elderly people with age-associated cognitive decline and/or mild depressive moods: a Kahoku Longitudinal Aging Study. Geriatr Gerontol Int. 2005;5:267-75.

184. O'Leary E, Sheedy G, O'Sullivan K, Thoresen C. Cork Older Adult Intervention Project: outcomes of a gestalt therapy group with older adults. Counselling Psychology Quarterly. 2003;16(2):131-43.

185. Oranta O, Luutonen S, Salokangas RK, Vahlberg T, Leino-Kilpi H. The outcomes of interpersonal counselling on depressive symptoms and distress after myocardial infarction. Nord J Psychiatry. 2010;64:78-86.

186. Oslin DW, Sayers S, Ross J, Kane V, Ten Have T, Conigliaro J, et al. Disease management for depression and at-risk drinking via telephone in an older population of veterans. Psychosom Med. 2003;65(6):931-7.

187. Pariser D, O'Hanlon A. Effects of telephone intervention on arthritis self-efficacy, depression, pain, and fatigue in older adults with arthritis. J Geriatr Phys Ther. 2005;28:67-73.

188. Pariser D, O'Hanlon AM, Speaker RS, Espinoza L. The effects of telephone intervention on arthritis self-efficacy, depression, pain and fatigue in older adults. Journal of Geriatric Physical Therapy. 2003;26:45-.

189. Patel V, Weiss HA, Chowdhary N, Naik S, Pednekar S, Chatterjee S, et al. Effectiveness of an intervention led by lay health counsellors for depressive and anxiety disorders in primary care in Goa, India (MANAS): a cluster randomised controlled trial. Lancet. 2010;376:2086-95.

190. Penckofer SM, Ferrans C, Mumby P, Byrn M, Emanuele MA, Harrison PR, et al. A psychoeducational intervention (SWEEP) for depressed women with diabetes. Ann Behav Med. 2012;44:192-206.

191. Pickett YR, Kennedy GJ, Freeman K, Cummings J, Woolis W. The effect of telephone-facilitated depression care on older, medically ill patients. J Behav Health Serv Res. 2014;41(1):90-6.

192. Popa-Velea O, Cernat B, Tambu A. Influence of personalized therapeutic approach on quality of life and psychiatric comorbidity in patients with advanced colonic cancer requiring palliative care. J Med Life. 2010;3:343-7.

193. Pot AM, Bohlmeijer ET, Onrust S, Melenhorst AS, Veerbeek M, De V. The impact of life review on depression in older adults: a randomized controlled trial. Int Psychogeriatr. 2010;22:572-81.

194. Pots WTM, Meulenbeek PAM, Veehof MM, Klungers J, Bohlmeijer ET. The efficacy of mindfulness-based cognitive therapy as a public mental health intervention for adults with mild to moderate depressive symptomatology: A randomized controlled trial. PLoS ONE. 2014;9(10).

195. Raglio A, Bellelli G, Traficante D, Gianotti M, Ubezio Maria C, Villani D, et al. Efficacy of music therapy in the treatment of behavioral and psychiatric symptoms of dementia. Alzheimer Dis Assoc Disord. 2008;22:158-62.

196. Raglio A, Oasi O, Gianotti M, Manzoni V, Bolis S, Ubezio MC, et al. Effects of music therapy on psychological symptoms and heart rate variability in patients with dementia. A pilot study. Curr Aging Sci. 2010;3:242-6.

197. Rawl SM, Given BA, Given CW, Champion VL, Kozachik SL, Kozachik SL, et al. Intervention to improve psychological functioning for newly diagnosed patients with cancer. Oncol Nurs Forum. 2002;29:967-75.

198. Reynolds CF, rd, Frank E, Kupfer DJ, Thase ME, Perel JM, et al. Treatment outcome in recurrent major depression: a post hoc comparison of elderly ("young old") and midlife patients. Am J Psychiatry. 1996;153:1288-92.

199. Reynolds CF, rd, Frank E, Perel JM, Imber SD, Cornes C, et al. Nortriptyline and interpersonal psychotherapy as maintenance therapies for recurrent major depression: a randomized controlled trial in patients older than 59 years. JAMA. 1999;281:39-45.

200. Reynolds CF, rd, Miller MD, Pasternak RE, Frank E, Perel JM, et al. Treatment of bereavement-related major depressive episodes in later life: a controlled study of acute and continuation treatment with nortriptyline and interpersonal psychotherapy. Am J Psychiatry. 1999;156:202-8.

201. Reynolds 3rd CF, Thomas SB, Morse JQ, Anderson SJ, Albert S, Dew MA, et al. Early intervention to preempt major depression among older black and white adults. Psychiatric Services. 2014;65(6):765-73.

202. Riegel B, Carlson B, Glaser D, Romero T. Randomized controlled trial of telephone case management in Hispanics of Mexican origin with heart failure. J Card Fail. 2006;12:211-9.

203. Risos-Rio GER. The effects of a structured live music and arts program on depression, pain control and mood/behavior patterns among nursing home residents. UPNAAI Nursing Journal. 2009;5:9-15.

204. Rizvi SJ, Zaretsky A, Schaffer A, Levitt A. Is immediate adjunctive CBT more beneficial than delayed CBT in treating depression? A pilot study. Journal of Psychiatric Practice. 2015;21:107-13.

205. Rohricht F, Papadopoulos N, Priebe S. An exploratory randomized controlled trial of body psychotherapy for patients with chronic depression. J Affect Disord. 2013;151(1):85-91.

206. Rokke PD, Tomhave JA, Jocic Z. The role of client choice and target selection in self-management therapy for depression in older adults. Psychology and aging. 1999;14:155-69.

207. Rokke PD, Tomhave JA, Jocic Z. Self-management therapy and educational group therapy for depressed elders. Cognitive Therapy & Research. 2000:99-119.

208. Rucci P, Frank E, Scocco P, Calugi S, Miniati M, Fagiolini A, et al. Treatment-emergent suicidal ideation during 4 months of acute management of unipolar major depression with SSRI pharmacotherapy or interpersonal psychotherapy in a randomized clinical trial. Depress Anxiety. 2011;28:303-9.

209. Sabir M, Henderson CR, Kang S-Y, Pillemer K. Attachment-focused integrative reminiscence with older African Americans: a randomized controlled intervention study. Aging & Mental Health. 2016;20(5):517-28.

210. Safren SA, Gonzalez JS, Wexler DJ, Psaros C, Delahanty LM, Blashill AJ, et al. A randomized controlled trial of cognitive behavioral therapy for adherence and depression (CBT-AD) in patients with uncontrolled type 2 diabetes. Diabetes Care. 2014;37(3):625-33.

211. Salminen M, Isoaho R, Vahlberg T, Ojanlatva A, Kivela SL. Effects of a health advocacy, counselling, and activation programme on depressive symptoms in older coronary heart disease patients. Int J Geriatr Psychiatry. 2005;20:552-8.

212. Schramm E, Zobel I, Dykierek P, Kech S, Brakemeier EL, Kulz A, et al. Cognitive behavioral analysis system of psychotherapy versus interpersonal psychotherapy for early-onset chronic depression: a randomized pilot study. J Affect Disord. 2011;129(1-3):109-16.

213. Scogin F, Hamblin D, Beutler L. Bibliotherapy for depressed older adults: a self-help alternative. Gerontologist. 1987; (3):383-7.

214. Scogin F, Jamison C, Davis N. Two-year follow-up of bibliotherapy for depression in older adults. Journal of Consulting and Clinical Psychology. 1990:665-7.

215. Scogin F, Jamison C, Gochneaur K. Comparative efficacy of cognitive and behavioral bibliotherapy for mildly and moderately depressed older adults. J Consult Clin Psychol. 1989;57(3):403-7.

216. Scogin FR, Moss K, Harris GM, Presnell AH. Treatment of depressive symptoms in diverse, rural, and vulnerable older adults. Int J Geriatr Psychiatry. 2014;29(3):310-6.

217. Scott J, Teasdale JD, Paykel ES, Johnson AL, Abbott R, Hayhurst H, et al. Effects of cognitive therapy on psychological symptoms and social functioning in residual depression. Br J Psychiatry. 2000;177:440-6.

218. Sebregts EH, Falger PR, Appels A, Kester AD, Bar FW. Psychological effects of a short behavior modification program in patients with acute myocardial infarction or coronary artery bypass grafting. A randomized controlled trial. J Psychosom Res. 2005;58:417-24.

219. Shellman JM, Mokel M, Hewitt N. The effects of integrative reminiscence on depressive symptoms in older African Americans. West J Nurs Res. 2009;31:772-86.

220. Simon GE, Ralston JD, Savarino J, Pabiniak C, Wentzel C, Operskalski BH. Randomized trial of depression follow-up care by online messaging. J Gen Intern Med. 2011;26:698-704.

221. Simpson GK, Tate RL, Whiting DL, Cotter RE. Suicide prevention after traumatic brain injury: a randomized controlled trial of a program for the psychological treatment of hopelessness. J Head Trauma Rehabil. 2011;26:290-300.

222. Simson U, Nawarotzky U, Friese G, Porck W, Schottenfeld-Naor Y, Hahn S, et al. Psychotherapy intervention to reduce depressive symptoms in patients with diabetic foot syndrome. Diabetic Medicine. 2008;25:206-12.

223. Sloman R. Relaxation and imagery for anxiety and depression control in community patients with advanced cancer. Cancer Nurs. 2002;25:432-5.

224. Snarski M, Scogin F, DiNapoli E, Presnell A, McAlpine J, Marcinak J. The effects of behavioral activation therapy with inpatient geriatric psychiatry patients. Behav Ther. 2011;42:100-8.

225. Spek V, Cuijpers P, Nyklicek I, Smits N, Riper H, Keyzer J, et al. One-year follow-up results of a randomized controlled clinical trial on internet-based cognitive behavioural therapy for subthreshold depression in people over 50 years. Psychol Med. 2008;38:635-9.

226. Spek V, Nyklicek I, Smits N, Cuijpers P, Riper H, Keyzer J, et al. Internet-based cognitive behavioural therapy for subthreshold depression in people over 50 years old: a randomized controlled clinical trial. Psychol Med. 2007;37:1797-806.

227. Stasi MF, Amati D, Costa C, Resta D, Senepa G, Scarafioiti C, et al. Pet-therapy: a trial for institutionalized frail elderly patients. Archives of gerontology and geriatrics Supplement. 2004:407-12.

228. Steidtmann D, Manber R, Arnow BA, Klein DN, Markowitz JC, Rothbaum BO, et al. Patient treatment preference as a predictor of response and attrition in treatment for chronic depression. Depress Anxiety. 2012;29:896-905.

229. Stevens-Ratchford RG. The effect of life review reminiscence activities on depression and self-esteem in older adults. Am J Occup Ther. 1993;47:413-20.

230. Stinson CK, Kirk E. Structured reminiscence: an intervention to decrease depression and increase self-transcendence in older women. J Clin Nurs. 2006;15:208-18.

231. Stinson CK, Young EA, Kirk E, Walker R. Use of a structured reminiscence protocol to decrease depression in older women. J Psychiatr Ment Health Nurs. 2010;17:665-73.

232. Strachowski D, Khaylis A, Conrad A, Neri E, Spiegel D, Taylor CB. The effects of cognitive behavior therapy on depression in older patients with cardiovascular risk. Depress Anxiety. 2007:E1-10.

233. Strong V, Waters R, Hibberd C, Murray G, Wall L, Walker J, et al. Management of depression for people with cancer (SMaRT oncology 1): a randomised trial. Lancet. 2008;372:40-8.

234. Su TW, Wu LL, Lin CP. The prevalence of dementia and depression in Taiwanese institutionalized leprosy patients, and the effectiveness evaluation of reminiscence therapy--a longitudinal, single-blind, randomized control study. Int J Geriatr Psychiatry. 2012;27:187-96.

235. Tang TZ, DeRubeis RJ, Hollon SD, Amsterdam J, Shelton R, Schalet B. Personality change during depression treatment: a placebo-controlled trial. Arch Gen Psychiatry. 2009;66:1322-30.

236. Taveira TH, Dooley AG, Cohen LB, Khatana SAM, Wu WC. Pharmacist-led group medical appointments for the management of type 2 diabetes with comorbid depression in older adults. Annals of Pharmacotherapy. 2011;45:1346-55.

237. Taylor-Price C. The efficacy of structured reminiscence group psychotherapy as an intervention to decrease depression and increase psychological well-being in female nursing home residents. US1996.

238. Thompson L, Gallagher D, Czirr R. Personality disorder and outcome in the treatment of late-life depression. Journal of Geriatric Psychiatry. 1988:133-46.

239. Thompson LW, Coon DW, Gallagher-Thompson D, Sommer BR, Koin D. Comparison of desipramine and cognitive/behavioral therapy in the treatment of elderly outpatients with mild-to-moderate depression. Am J Geriatr Psychiatry. 2001;9(3):225-40.

240. Thompson LW, Gallagher D. Efficacy of psychotherapy in the treatment of late-life depression. Advances in Behaviour Research & Therapy. 1984:127-39.

241. Thompson LW, Gallagher D, Breckenridge JS. Comparative effectiveness of psychotherapies for depressed elders. Journal of Consulting and Clinical Psychology. 1987; (3):385-90.

242. Thompson NJ, Patel AH, Selwa LM, Stoll SC, Begley CE, Johnson EK, et al. Expanding the efficacy of project UPLIFT: Distance delivery of mindfulness-based depression prevention to people with epilepsy. Journal of Consulting and Clinical Psychology. 2015;83(2):304-13.

243. Titov N, Dear BF, Ali S, Zou JB, Lorian CN, Johnston L, et al. Clinical and Cost-Effectiveness of Therapist-Guided Internet-Delivered Cognitive Behavior Therapy for Older Adults With Symptoms of Depression: A Randomized Controlled Trial. Behavior Therapy. 2015;46(2):193-205.

244. Tovote KA, Fleer J, Snippe E, Peeters AC, Emmelkamp PM, Sanderman R, et al. Individual mindfulness-based cognitive therapy and cognitive behavior therapy for treating depressive symptoms in patients with diabetes: results of a randomized controlled trial. Diabetes Care. 2014;37(9):2427-34.

245. Tsai HH, Tsai YF. Changes in depressive symptoms, social support, and loneliness over 1 year after a minimum 3-month videoconference program for older nursing home resident. J Med Internet Res. 2011; (4):373-84.

246. Tsai H-H, Tsai Y-F, Wang H-H, Chang Y-C, Chu Hao H. Videoconference program enhances social support, loneliness, and depressive status of elderly nursing home residents. Aging Ment Health. 2010;14:947-54.

247. Turner A, Hambridge J, Baker A, Bowman J, McElduff P. Randomised controlled trial of group cognitive behaviour therapy versus brief intervention for depression in cardiac patients. The Australian and New Zealand journal of psychiatry. 2013;47:235-43.

248. Turner A, Murphy BM, Higgins RO, Elliott PC, Le Grande MR, Goble AJ, et al. An integrated secondary prevention group programme reduces depression in cardiac patients. European journal of preventive cardiology. 2014;21(2):153-62.

249. Tyrer P, Jones V, Thompson S, Catalan J, Schmidt U, Davidson K, et al. Service variation in baseline variables and prediction of risk in a randomised controlled trial of psychological treatment in repeated parasuide: The Popmact study. International Journal of Social Psychiatry. 2003;49:58-69.

250. Wagner B, Horn AB, Maercker A. Internet-based versus face-to-face cognitive-behavioral intervention for depression: A randomized controlled non-inferiority trial. Journal of Affective Disorders. 2014;152-154(1):113-21.

251. Van Beljouw IMJ, Van Exel E, Van De Ven PM, Joling KJ, Dhondt TDF, Stek ML, et al. Does an outreaching stepped care program reduce depressive symptoms in community-dwelling older adults? A randomized implementation trial. American Journal of Geriatric Psychiatry. 2015;23(8):807-17.

252. Van Der M, I C, May AM, Ros WJG, Oosterom M, Hordijk GJ, et al. One-year effect of a nurse-led psychosocial intervention on depressive symptoms in patients with head and neck cancer: A randomized controlled trial. Oncologist. 2013;18:336-44.

253. van S, van M, Ader H, van D, de H, Penninx B, et al. Interpersonal psychotherapy for elderly patients in primary care. Am J Geriatr Psychiatry. 2006;14:777-86.

254. Wang JJ, Hsu YC, Cheng SF. The effects of reminiscence in promoting mental health of Taiwanese elderly. Int J Nurs Stud. 2005;42:31-6.

255. Wang JJ. Group reminiscence therapy for cognitive and affective function of demented elderly in Taiwan. Int J Geriatr Psychiatry. 2007;22:1235-40.

256. Wang J. The effects of reminiscence on depressive symptoms and mood status of older institutionalized adults in Taiwan. Int J Geriatr Psychiatry. 2005;20:57-62.

257. Wang JJ, Lin YH, Hsieh LY. Effects of gerotranscendence support group on gerotranscendence perspective, depression, and life satisfaction of institutionalized elders. Aging Ment Health. 2011;15:580-6.

258. Wang L, Li J. Role of educational intervention in the management of comorbid depression and hypertension. Blood Press. 2003;12:198-202.

259. Warber SL, Ingerman S, Moura VL, Wunder J, Northrop A, Gillespie BW, et al. Healing the heart: a randomized pilot study of a spiritual retreat for depression in acute coronary syndrome patients. Explore (NY). 2011;7:222-33.

260. Watt LM, Cappeliez P. Integrative and instrumental reminiscence therapies for depression in older adults: intervention strategies and treatment effectiveness. Aging Ment Health. 2000;4:166-77.

261. Weber BA, Roberts BL, Yarandi H, Mills TL, Chumbler NR, Wajsman Z. The impact of dyadic social support on self-efficacy and depression after radical prostatectomy. J Aging Health. 2007;19:630-45.

262. Werneck AL, Rosso AL, Vincent MB. The use of an antagonist 5-HT2a/c for depression and motor function in Parkinson' disease. Arq Neuropsiquiatr. 2009;67:407-12.

263. Westerhof GJ, Bohlmeijer ET, van B, I M, Pot AM. Improvement in personal meaning mediates the effects of a life review intervention on depressive symptoms in a randomized controlled trial. Gerontologist. 2010;50:541-9.

264. Wetherell Julie L, Ayers Catherine R, Sorrell John T, Thorp Steven R, Nuevo R, Belding W, et al. Modular psychotherapy for anxiety in older primary care patients. The American Journal of Geriatric Psychiatry. 2009;17:483-92.

265. White H, McConnell E, Clipp E, Branch LG, Sloane R, Pieper C, et al. A randomized controlled trial of the psychosocial impact of providing internet training and access to older adults. Aging Ment Health. 2002;6:213-21.

266. Willemse GR, Smit F, Cuijpers P, Tiemens BG. Minimal-contact psychotherapy for sub-threshold depression in primary care. Randomised trial. Br J Psychiatry. 2004;185:416-21.

267. Williams JM, Crane C, Barnhofer T, Brennan K, Duggan DS, Fennell MJ, et al. Mindfulness-based cognitive therapy for preventing relapse in recurrent depression: a randomized dismantling trial. J Consult Clin Psychol. 2014;82(2):275-86.

268. Wolinsky FD, Vander W, M W, Martin R, Unverzagt FW, Ball KK, et al. The effect of speed-of-processing training on depressive symptoms in ACTIVE. The journals of gerontology Series A, Biological sciences and medical sciences. 2009;64:468-72.

269. Wolinsky FD, Vander Weg MW, Howren MB, Jones MP, Dotson MM. The Effect of Cognitive Speed of Processing Training on the Development of Additional IADL Difficulties and the Reduction of Depressive Symptoms: Results From the IHAMS Randomized Controlled Trial. Journal of Aging & Health. 2015;27(2):334-54.

270. Worley MJ, Trim RS, Tate SR, Hall JE, Brown SA. Service utilization during and after outpatient treatment for comorbid substance use disorder and depression. J Subst Abuse Treat. 2010;39:124-31.

271. Vos PJ, Visser AP, Garssen B, Duivenvoorden HJ, de H, H C. Effectiveness of group psychotherapy compared to social support groups in patients with primary, non-metastatic breast cancer. J Psychosoc Oncol. 2007;25:37-60.

272. Wurtzen H, Dalton SO, Elsass P, Sumbundu AD, Steding-Jensen M, Karlsen RV, et al. Mindfulness significantly reduces self-reported levels of anxiety and depression: Results of a randomised controlled trial among 336 Danish women treated for stage I-III breast cancer. European Journal of Cancer. 2013;49:1365-73.

273. Wuthrich VM, Rapee RM. Randomised controlled trial of group cognitive behavioural therapy for comorbid anxiety and depression in older adults. Behav Res Ther. 2013;51(12):779-86.

274. Young Laura A, Baime Michael J. Mindfulness-based stress reduction: Effect on emotional distress in older adults. Complementary Health Practice Review. 2010;15:59-64.

275. Zerhusen JD, Boyle K, Wilson W. Out of the darkness: group cognitive therapy for depressed elderly. Journal of psychosocial nursing and mental health services. 1991:16-21.

276. Zhou KN, Li XM, Yan H, Dang SN, Wang DL. Effects of music therapy on depression and duration of hospital stay of breast cancer patients after radical mastectomy. Chin Med J (Engl). 2011;124:2321-7.

277. Zhou W, He G, Gao J, Yuan Q, Feng H, Zhang CK. The effects of group reminiscence therapy on depression, self-esteem, and affect balance of Chinese community-dwelling elderly. Arch Gerontol Geriatr. 2012;54:e440-7.

Intervention

1. Chan MF, Leong KS, Heng BL, Mathew BK, Khan SB, Lourdusamy SS, et al. Reducing depression among community-dwelling older adults using life-story review: a pilot study. Geriatric nursing. 2014;35(2):105-10.

2. Cartwright M, Hirani SP, Rixon L, Beynon M, Doll H, Bower P, et al. Effect of telehealth on quality of life and psychological outcomes over 12 months (Whole Systems Demonstrator telehealth questionnaire study): nested study of patient reported outcomes in a pragmatic, cluster randomised controlled trial. BMJ (Clinical research ed). 2013;346:f653.

3. Cole Martin G, Fenton Fred R, Engelsmann F, Mansouri I. Effectiveness of geriatric psychiatry consultation in an acute care hospital: A randomized clinical trial. J Am Geriatr Soc. 1991;39:1183-8.

4. Gask L, Dowrick C, Dixon C, Sutton C, Perry R, Torgerson D, et al. A pragmatic cluster randomized controlled trial of an educational intervention for GPs in the assessment and management of depression. Psychol Med. 2004;34:63-72.

5. Imai H, Furukawa TA, Okumiya K, Wada T, Fukutomi E, Sakamoto R, et al. Postcard intervention for depression in community-dwelling older adults: A randomised controlled trial. Psychiatry Research. 2015;229.545-50.

6. Latour D, Cappeliez P. Pretherapy training for group cognitive therapy with depressed older adults. Canadian Journal on Aging. 1994;13:221-35.

7. Liu X, Niu X, Feng Q, Liu Y. Effects of five-element music therapy on elderly people with seasonal affective disorder in a Chinese nursing home. Journal of traditional Chinese medicine = Chung i tsa chih ying wen pan / sponsored by All-China Association of Traditional Chinese Medicine, Academy of Traditional Chinese Medicine. 2014;34(2):159-61.

8. McPaul J. Focus on research... Home visit versus telephone follow-up in Phase II cardiac rehabilitation following myocardial infarction: effects on anxiety, depression, attendance at Phase III and visits to A&E or readmission. British Journal of Occupational Therapy. 2008;71:320-.

9. Nygaard B, Jensen EW, Kvetny J, Jarlov A, Faber J. Effect of combination therapy with thyroxine (T4) and 3,5,3'-triiodothyronine versus T4 monotherapy in patients with hypothyroidism, a double-blind, randomised cross-over study. European journal of endocrinology / European Federation of Endocrine Societies. 2009;161(6):895-902.

10. Sherrill JT, Frank E, Geary M, Stack JA, Reynolds CF, rd. Psychoeducational workshops for elderly patients with recurrent major depression and their families. Psychiatr Serv. 1997;48:76-81.

11. Waterreus A, Blanchard M, Mann A. Community psychiatric nurses for the elderly: well tolerated, few side-effects and effective in the treatment of depression. J Clin Nurs [Internet]. 1994; (5):299-306.

12. Wilkinson P, Alder N, Juszczak E, Matthews H, Merritt C, Montgomery H, et al. A pilot randomised controlled trial of a brief cognitive behavioural group intervention to reduce recurrence rates in late life depression. Int J Geriatr Psychiatry. 2009;24:68-75.

Outcome

1. Alexopoulos GS, Raue PJ, Kiosses DN, Seirup JK, Banerjee S, Arean PA. Comparing engage with PST in late-life major depression: A preliminary report. American Journal of Geriatric Psychiatry. 2015;23(5):506-13.

2. Berger AM, Wielgus K, Hertzog M, Fischer P, Farr L. Patterns of circadian activity rhythms and their relationships with fatigue and anxiety/depression in women treated with breast cancer adjuvant chemotherapy. Support Care Cancer. 2010;18:105-14.

3. Korte J, Cappeliez P, Bohlmeijer Ernst T, Westerhof Gerben J. Meaning in life and mastery mediate the relationship of negative reminiscence with psychological distress among older adults with mild to moderate depressive symptoms. European Journal of Ageing. 2012;9:343-51.

4. Marmar CR, Gaston L, Gallagher D, Thompson LW. Alliance and outcome in late-life depression. J Nerv Ment Dis. 1989;177(8):464-72.

5. McCaffrey R. The effect of healing gardens and art therapy on older adults with mild to moderate depression. Holistic Nursing Practice. 2007;21(2):79-84.

6. Romeo R, Knapp M, Banerjee S, Morris J, Baldwin R, Tarrier N, et al. Treatment and prevention of depression after surgery for hip fracture in older people: cost-effectiveness analysis. J Affect Disord. 2011;128:211-9.

7. Schaik v, D J, van M, H W, Beekman AT, de H, et al. Interpersonal psychotherapy (IPT) for late-life depression in general practice: uptake and satisfaction by patients, therapists and physicians. BMC Fam Pract. 2007;8:52.

8. Unutzer J, Katon WJ, Fan MY, Schoenbaum MC, Lin EH, Della P, et al. Long-term cost effects of collaborative care for late-life depression. Am J Manag Care. 2008;14:95-100.

Study design

1. Afonso Rosa M, Bueno B, Loureiro Manuel J, Pereira H. Reminiscence, Psychological Well-Being, and Ego Integrity in Portuguese Elderly People. Educational Gerontology. 2011;37:1063-80.

2. Bilotta C, Bergamaschini L, Spreafico S, Vergani C. Day care centre attendance and quality of life in depressed older adults living in the community. European Journal of Ageing. 2010;7(1):29-35.

3. Bohlmeijer ET, Westerhof GJ, Emmerik-de J. The effects of integrative reminiscence on meaning in life: results of a quasi-experimental study. Aging Ment Health. 2008;12:639-46.

4. Bosma H, Lamers F, Jonkers CC, van Eijk JT. Disparities by education level in outcomes of a self-management intervention: the DELTA trial in The Netherlands. Psychiatr Serv. 2011;62(7):793-5.

5. Choi NG, Hegel MT, Sirrianni L, Marinucci ML, Bruce ML. Passive coping response to depressive symptoms among low-income homebound older adults: does it affect depression severity and treatment outcome? Behav Res Ther. 2012;50:668-74.

6. Dakin EK, Arean P. Patient perspectives on the benefits of psychotherapy for late-life depression. American Journal of Geriatric Psychiatry. 2013;21:155-63.

7. Delaney C, Apostolidis B. Pilot testing of a multicomponent home care intervention for older adults with heart failure: an academic clinical partnership. J Cardiovasc Nurs. 2010:E27-40.

8. Donkin L, Hickie IB, Christensen H, Naismith SL, Neal B, Cockayne NL, et al. Sampling bias in an internet treatment trial for depression. Transl Psychiatry. 2012;2:e174.

9. Ernst S, Welke J, Heintze C, Gabriel R, Zollner A, Kiehne S, et al. Effects of mindfulness-based stress reduction on quality of life in nursing home residents: a feasibility study. Forsch Komplementmed. 2008;15:74-81.

10. Flemming N, Kjeld A, Lolk A. Unipolar depression in elderly treated with home visits: Follow-up study. Neuroepidemiology. 2012;39:225-6.

11. Haringsma R, Engels GI, Spinhoven P. Predictors of response to the 'Coping with Depression' course for older adults. A field study. Aging Ment Health. 2006;10:424-34.

12. Harrison M, Reeves D, Harkness E, Valderas J, Kennedy A, Rogers A, et al. A secondary analysis of the moderating effects of depression and multimorbidity on the effectiveness of a chronic disease self-management programme. Patient Educ Couns. 2012;87:67-73.

13. Hedman E, Ljotsson B, Kaldo V, Hesser H, El Alaoui S, Kraepelien M, et al. Effectiveness of Internet-based cognitive behaviour therapy for depression in routine psychiatric care. J Affect Disord. 2014;155:49-58.

14. Hsu CT, Weng CY, Kuo CS, Lin CL, Jong MC, Kuo SY, et al. Effects of a cognitive-behavioral group program for community-dwelling elderly with minor depression. Int J Geriatr Psychiatry. 2010:654-5.

15. Jones ED. Reminiscence therapy for older women with depression. Effects of nursing intervention classification in assisted-living long-term care. J Gerontol Nurs. 2003;29:26-33; quiz 56.

16. Kiosses DN, Rosenberg PB, McGovern A, Fonzetti P, Zaydens H, Alexopoulos GS. Depression and Suicidal Ideation During Two Psychosocial Treatments in Older Adults with Major Depression and Dementia. Journal of Alzheimer's Disease. 2015;48.453-62.

17. Koenig AM, Jarrett RB, Gallop R, Barrett MS, Thase ME. Extreme nonresponse to acute phase cognitive therapy for depression: An attempt to replicate and extend. Behavior Therapy. 2014;45(3):300-13.

18. Korte J, Bohlmeijer ET, Cappeliez P, Smit F, Westerhof GJ. Life review therapy for older adults with moderate depressive symptomatology: a pragmatic randomized controlled trial. Psychol Med. 2012;42:1163-73.

18. Mastel-Smith B, Binder B, Malecha A, Hersch G, Symes L, McFarlane J. Testing therapeutic life review offered by home care workers to decrease depression among home-dwelling older women. Issues Ment Health Nurs. 2006;27:1037-49.

20. McMurchie W, Macleod F, Power K, Laidlaw K, Prentice N. Computerised cognitive behavioural therapy for depression and anxiety with older people: A pilot study to examine patient acceptability and treatment outcome. Int J Geriatr Psychiatry. 2013.

21. Mewton L, Andrews G. Cognitive behaviour therapy via the internet for depression: A useful strategy to reduce suicidal ideation. Journal of Affective Disorders. 2015;170:78-84.

22. Miller MD, Frank E, Cornes C, Houck PR, Reynolds CF, rd. The value of maintenance interpersonal psychotherapy (IPT) in older adults with different IPT foci. Am J Geriatr Psychiatry. 2003;11:97-102.

23. Morgan AJ, Mackinnon AJ, Jorm AF. Behavior change through automated e-mails: Mediation analysis of self-help strategy use for depressive symptoms. Behav Res Ther. 2013;51(2):57-62.

24. O’Connor M, Piet J, Hougaard E. The effects of mindfulness-based cognitive therapy on depressive symptoms in elderly bereaved people with loss-related distress: A controlled pilot study. Mindfulness. 2014;5:400-9.

25. Oxman TE, Hull JG. Social support and treatment response in older depressed primary care patients. J Gerontol B Psychol Sci Soc Sci. 2001;56:P35-45.

26. Oyama H, Ono Y, Watanabe N, Tanaka E, Kudoh S, Sakashita T, et al. Local community intervention through depression screening and group activity for elderly suicide prevention. Psychiatry Clin Neurosci. 2006:110-4.

27. Raue PJ, Morales KH, Post EP, Bogner HR, Have TT, Bruce ML. The wish to die and 5-year mortality in elderly primary care patients. Am J Geriatr Psychiatry. 2010;18:341-50.

28. Rosenthal MZ, Cheavens JS, Compton JS, Thorp SR, Lynch TR. Thought suppression and treatment outcome in late-life depression. Aging and Mental Health. 2005;9:35-9.

29. Serfaty M, Csipke E, Haworth D, Murad S, King M. A talking control for use in evaluating the effectiveness of cognitive-behavioral therapy. Behav Res Ther. 2011;49:433-40.

30. Steuer JL, Mintz J, Hammen CL, Hill MA, Jarvik LF, McCarley T, et al. Cognitive-behavioral and psychodynamic group psychotherapy in treatment of geriatric depression. Journal of Consulting and Clinical Psychology [Internet]. 1984; (2):180-9.

31. Thomas SA, Lincoln NB. Factors relating to depression after stroke. Br J Clin Psychol. 2006;45:49-61.

32. Verkaik R, Francke AL, van M, Spreeuwenberg PM, Ribbe MW, Bensing JM. The effects of a nursing guideline on depression in psychogeriatric nursing home residents with dementia. Int J Geriatr Psychiatry. 2011;26:723-32.

33. Youssef FA. The impact of group reminiscence counseling on a depressed elderly population. Nurse Pract. 1990;15:32, 5-8.

Type of publication

1. Home-based programme significantly reduces depressive symptoms and improves health status in chronically ill older adults with minor depression or dysthymia. Evidence-Based Healthcare and Public Health. 2004;8:257-8.

2. Cognitive behavioral therapy effective for depressed elderly. J Natl Med Assoc. 2010;102(4):358.

3. Beary T. Living with the 'black dog': depression in care homes. Nursing & Residential Care. 2013;15:83-7.

4. Buist-Bouwman MA. Collaborative care management improves physical functioning in older people with depression. Evidence Based Mental Health. 2005;8(4):106-.

5. Carreira K, Miller MD, Frank E, Houck PR, Morse JQ, Dew MA, et al. A controlled evaluation of monthly maintenance interpersonal psychotherapy in late-life depression with varying levels of cognitive function. Int J Geriatr Psychiatry. 2008;23(11):1110-3.

6. Chou KR, Lin Y. The effectiveness of group music therapy to improve depression and cognition status in elderly persons with dementia. European Psychiatry. 2012;27.

7. Cuijpers P, Karyotaki E, Pot AM, Park M, Reynolds CF. Managing depression in older age: Psychological interventions. Maturitas. 2014;79(2):160-9.

8. de M, Saskia, Dekker J, Schoevers R, van A, Gerda, et al. Short psychodynamic supportive psychotherapy, antidepressants, and their combination in the treatment of major depression: A mega-analysis based on three randomized clinical trials. Depress Anxiety. 2008;25:565-74.

9. Freedland KE, Carney RM, Hayano J, Steinmeyer BC, Reese RL, Roest AM. Effect of obstructive sleep apnea on response to cognitive behavior therapy for depression after an acute myocardial infarction. J Psychosom Res. 2012;72:276-81.

10. Furukawa TA. A stepped-care prevention programme decreased depression and anxiety disorders in elderly patients. Evidence Based Medicine. 2009;14:146-.

11. Gallagher DE. Comparative effectiveness of group psychotherapies for reduction of depression in elderly outpatients. Dissertation Abstracts International [Internet]. 1979; (11-b):5550-1.

12. Gallagher DE, Thompson LW. Treatment of major depressive disorder in older adult outpatients with brief psychotherapies. Psychotherapy (Chicago, Ill). 1982:482-90.

13. Garland EL. [Commentary On] Dismantling mindfulness-based cognitive therapy for recurrent depression implicates lack of differential efficacy for mindfulness training. Evidence Based Mental Health. 2014;17(3):94-.

14. Gatz M, Fiske A, Fox Lauren S, Kaskie B, Kasl-Godley Julia E, McCallum Todd J, et al. Empirically validated psychological treatments for older adults. Journal of Mental Health and Aging. 1998;4:9-46.

15. Geron SM, Keefe B. Moving evidence-based interventions to populations: a case study using social workers in primary care. Home Health Care Serv Q. 2006;25:95-113.

16. Gilbody S. Depression in older adults: collaborative care model seems effective. Evidence Based Mental Health. 2008;11(2):44-.

17. Gitlin LN, Harris LF, McCoy M, Chernett NL, Jutkowitz E, Pizzi LT, et al. A community-integrated home based depression intervention for older African Americans: [corrected] description of the Beat the Blues randomized trial and intervention costs. BMC geriatrics. 2012;12:4.

18. Gum AM, Arean PA, Bostrom A. Low-income depressed older adults with psychiatric comorbidity: secondary analyses of response to psychotherapy and case management. Int J Geriatr Psychiatry. 2007;22:124-30.

19. Hansson M, Bodlund O, Chotai J. Patient education and group counselling to improve the treatment of depression in primary care: a randomized controlled trial. J Affect Disord. 2008;105:235-40.

20. Holman AJ, Serfaty MA, Leurent BE, King MB. Cost-effectiveness of cognitive behaviour therapy versus talking and usual care for depressed older people in primary care. BMC health services research. 2011;11:33.

21. Hummel J, Weisbrod C, Oster P, Kopf D. The effect of cognitive behavioral therapy in multimorbid geriatric patients with depression. European Geriatric Medicine. 2013;4:S203.

22. Jonkers CC, Lamers F, Evers SM, Bosma H, Metsemakers JF, Van Eijk JT. Economic evaluation of a minimal psychological intervention in chronically ill elderly patients with minor or mild to moderate depression: a randomized trial (the DELTA-study). International journal of technology assessment in health care. 2009;25(4):497-504.

23. Knapp P, Young J, House A, Forster A. Non-drug strategies to resolve psycho-social difficulties after stroke. Age Ageing. 2000;29:23-30.

24. Kohen R, Cain KC, Buzaitis A, Johnson V, Becker KJ, Teri L, et al. Response to psychosocial treatment in poststroke depression is associated with serotonin transporter polymorphisms. Stroke. 2011;42:2068-70.

25. Kootker JA, Fasotti L, Rasquin SM, van H, C M, Geurts A. The effectiveness of an augmented cognitive behavioural intervention for post-stroke depression with or without anxiety (PSDA): the Restore4Stroke-PSDA trial. BMC Neurol. 2012;12:51.

26. Kramer TL, Beaudin CL, Thrush CR. Evaluation and treatment of depression (part I): benefits for patients, providers, and payors. Disease Management & Health Outcomes. 2005;13:295-306.

27. Krieger T, Meyer B, Sude K, Urech A, Maercker A, Berger T. Evaluating an e-mental health program ("deprexis") as adjunctive treatment tool in psychotherapy for depression: design of a pragmatic randomized controlled trial. BMC Psychiatry. 2014;14:285.

28. Kuharek JM. Behavioral group therapy on depressed inpatient elderly: A comparative study. Dissertation Abstracts International. 1991; (1-a):265-6.

29. Laidlaw K. A randomised controlled trial of cognitive behaviour therapy versus treatment ad usual in the treatment of mild to moderate late life depression. National Research Register. 2002.

30. Lamers F, Jonkers CC, Bosma H, Diederiks JP, van E, J T. Effectiveness and cost-effectiveness of a minimal psychological intervention to reduce non-severe depression in chronically ill elderly patients: the design of a randomised controlled trial [ISRCTN92331982]. BMC public health. 2006;6:161.

31. Lynch TR. Treatment of elderly depression with personality disorder comorbidity using dialectical behavior therapy. Cognitive and Behavioral Practice. 2000:468-77.

32. Lyness JM, Heo M, Datto CJ, Ten H, T R, Katz IR, et al. Outcomes of minor and subsyndromal depression among elderly patients in primary care settings. Ann Intern Med. 2006;144:496-504.

33. Mastel-Smith B. An intervention to improve the health of people 60 years and older 2004.

34. Matlock JA. The impact of supportive therapy with geriatric patients in the first thirty days of nursing home admission 1995.

35. McCusker J, Cole M, Keller E, Bellavance F, Berard A, Jacoby R. Review: Heterocyclic antidepressants and rational psychological therapies reduce depression in older ambulatory patients. Evidence-Based Medicine. 1998;3:152.

36. McMillan D, Gilbody S. A stepped care intervention is effective for elderly people with subthreshold depression or anxiety. Evidence Based Mental Health. 2009;12:115-.

37. Overend K, Lewis H, Bailey D, Bosanquet K, Chew-Graham C, Ekers D, et al. CASPER plus (CollAborative care in Screen-Positive EldeRs with major depressive disorder): study protocol for a randomised controlled trial. Trials. 2014;15:451.

38. Patel V, Weobong B, Nadkarni A, Weiss HA, Anand A, Naik S, et al. The effectiveness and cost-effectiveness of lay counsellor-delivered psychological treatments for harmful and dependent drinking and moderate to severe depression in primary care in India: PREMIUM study protocol for randomized controlled trials. Trials. 2014;15:101.

39. Post EP, Miller MD, Schulberg HC. Using interpersonal therapy (IPT) to treat depression in older primary care patients. Geriatrics. 2008;63:18.

40. Scazufca M, Matsuda CMCB. Review of the efficacy of psychotherapy vs. pharmacotherapy for depression treatment in old age. Revista Brasileira de Psiquiatria. 2002;24:64-9.

41. Thielke SM, Fan MY, Sullivan M, Unutzer J. Pain limits the effectiveness of collaborative care for depression. Am J Geriatr Psychiatry. 2007;15:699-707.

42. Serfaty MA, Deborah H, Buszewicz M, Blanchard M, Murad S, King M. The clinical effectiveness of individual cognitive behaviour therapy for depressed older people in primary care and the use of a talking control (TC). European Psychiatry. 2011;26.

43. Simon G. In treatment-resistant depression, adding cognitive–behavioral therapy to usual care was cost-effective at 1 y. ACP Journal Club. 2014;160(11):1-.

44. Simon SS, Cordás TA, Bottino CMC. Cognitive behavioral therapies in older adults with depression and cognitive deficits: A systematic review. International Journal of Geriatric Psychiatry. 2015;30:223-33.

Language

1. Afonso R, Bueno B. [Reminiscence with different types of autobiographical memories: Effects on the reduction of depressive symptomatology in old age]. Psicothema. 2010;22:213-20.

2. Fan XY. Effect of psychologic interference on gastric power of sufferers from functional indigestion and depression. Chinese Journal of Clinical Rehabilitation. 2006;10:22-4.

3. Fernandez-Calvo B, Rodriguez-Perez R, Contador I, Rubio-Santorum A, Ramos F. [Efficacy of cognitive training programs based on new software technologies in patients with Alzheimer-type dementia]. Psicothema. 2011;23:44-50.

4. Guan WB, Gao DJ, Li AM, Ouyang S, Dai ZC. Recent effects of early psychological intervention for patients with post-stroke depression. Chinese Journal of Clinical Rehabilitation. 2004;8:6832-3.

5. Hautzinger M. Behavior therapy for depression in the elderly. Verhaltenstherapie [Internet]. 1992; (3):217-21 pp.

6. Hautzinger M, Welz S. Cognitive behavioral therapy for depressed elderly outpatients - A controlled randomized trial. Verhaltenstherapie & Psychosoziale Praxis [Internet]. 2004; (4):789-98.

7. Hautzinger M, Welz S. [Cognitive behavioral therapy for depressed older outpatients--a controlled, randomized trial]. Z Gerontol Geriatr. 2004;37(6):427-35.

8. Hautzinger M, Welz S. Kognitive Verhaltenstherapie bei Depressionen im Alter: Ergebnisse einer kontrollierten Vergleichsstudie unter ambulanten Bedingungen an Depressionen mittleren Schweregrads. Z Gerontol Geriatr. 2004;37:427-35.

9. Hautzinger M, Welz S. Kurz- und längerfristige wirksamkeit psychologischer interventionen bei depressionen im alter. Zeitschrift für Klinische Psychologie und Psychotherapie: Forschung und Praxis. 2008;37:52-60.

10. Hautzinger M, Welz S. Short- and long-term efficacy of psychological intervention for depression in older adults. [German]. [References]. Zeitschrift für Klinische Psychologie und Psychotherapie: Forschung und Praxis. 2008:52-60.

11. Hirsch RD, Junglas K, Konradt B, Jonitz MF. [Humor therapy in the depressed elderly: results of an empirical study]. Zeitschrift für Gerontologie und Geriatrie : Organ der Deutschen Gesellschaft für Gerontologie und Geriatrie. 2010:42-52.

12. Liu SK, Zhao XM, Xi ZM. [Incidence rate and acupuncture-moxibustion treatment of post-stroke depression]. Zhongguo Zhen Jiu. 2006;26:472-4.

13. Nomura N. [Individual reminiscence therapy improves self-esteem for Japanese community-dwelling older adults]. Shinrigaku Kenkyu. 2009;80:42-7.

14. Serrano JP, Latorre JM, Montan~es J. [Life review therapy based on retrieval of specific autobiographical memories in elderly individuals with depression]. Rev Esp Geriatr Gerontol. 2005:220-7.

15. Su XL, Xiao XC. Effect of psychotherapy on the motor functional rehabilitation in patients with post-stroke depression. Chinese Journal of Clinical Rehabilitation. 2004;8:3720-1.

16. van 't V-T, P J, Cuijpers P, Beekman AJ. [Prevention of depression and anxiety in older people]. Tijdschr Psychiatr. 2011;53:579-84.

17. Wu HS, Xu Y, Xu YF, Zhu HX, Zhu LP, Xue JJ. Effectiveness of case management of chronic disease for elderly depression in community [Chinese]. Chinese Mental Health Journal. 2010:245-9.

Duplicates

1. Gellis Zvi D, McGinty J, Tierney L, Jordan C, Burton J, Misener E. Randomized controlled trial of problem-solving therapy for minor depression in home care. Research on Social Work Practice. 2008;18:596-606.

Excluded publications – cost-effectiveness

Population

1. Alves J, Alves-Costa F, Magalhaes R, Goncalves OF, Sampaio A. Cognitive stimulation for Portuguese older adults with cognitive impairment: A randomized controlled trial of efficacy, comparative duration, feasibility, and experiential relevance. American Journal of Alzheimer's Disease and Other Dementias. 2014;29(6):503-12.

2. Apostolo JL, Cardoso DF, Rosa AI, Paul C. The effect of cognitive stimulation on nursing home elders: a randomized controlled trial. J Nurs Scholarsh. 2014;46(3):157-66.

3. Araya R, Flynn T, Rojas G, Fritsch R, Simon G. Cost-effectiveness of a primary care treatment program for depression in low-income women in Santiago, Chile. Am J Psychiatry. 2006;163(8):1379-87.

4. Barley EA, Walters P, Haddad M, Phillips R, Achilla E, McCrone P, et al. The UPBEAT nurse-delivered personalized care intervention for people with coronary heart disease who report current chest pain and depression: A randomised controlled pilot study. PLoS ONE. 2014;9(6).

5. Borghi J, Guest JF. Economic impact of using mirtazapine compared to amitriptyline and fluoxetine in the treatment of moderate and severe depression in the UK. Eur Psychiatry. 2000;15(6):378-87.

6. Bosmans J, de Bruijne M, van Hout H, van Marwijk H, Beekman A, Bouter L, et al. Cost-effectiveness of a disease management program for major depression in elderly primary care patients. J Gen Intern Med. 2006;21(10):1020-6.

7. Bosmans JE, Dozeman E, van Marwijk HW, van Schaik DJ, Stek ML, Beekman AT, et al. Cost-effectiveness of a stepped care programme to prevent depression and anxiety in residents in homes for the older people: a randomised controlled trial. Int J Geriatr Psychiatry. 2014;29(2):182-90.

8. Bosmans JE, Schreuders B, van Marwijk HW, Smit JH, van Oppen P, van Tulder MW. Cost-effectiveness of problem-solving treatment in comparison with usual care for primary care patients with mental health problems: a randomized trial. BMC Fam Pract. 2012;13:98.

9. Bosmans JE, van Schaik DJ, Heymans MW, van Marwijk HW, van Hout HP, de Bruijne MC. Cost-effectiveness of interpersonal psychotherapy for elderly primary care patients with major depression. International journal of technology assessment in health care. 2007;23(4):480-7.

10. Boyer P, Danion JM, Bisserbe JC, Hotton JM, Troy S. Clinical and economic comparison of sertraline and fluoxetine in the treatment of depression. A 6-month double-blind study in a primary-care setting in France. PharmacoEconomics. 1998;13(1 Pt 2):157-69.

11. Brown MCJ, Nimmerrichter AA, Guest JF. Cost-effectiveness of mirtazapine compared to amitriptyline and fluoxetine in the treatment of moderate and severe depression in Austria. European Psychiatry. 1999;14(4):230-44.

12. Browne G, Steiner M, Roberts J, Gafni A, Byrne C, Dunn E, et al. Sertraline and/or interpersonal psychotherapy for patients with dysthymic disorder in primary care: 6-month comparison with longitudinal 2-year follow-up of effectiveness and costs. J Affect Disord. 2002;68(2-3):317-30.

13. Chisholm D, Sanderson K, Ayuso-Mateos JL, Saxena S. Reducing the global burden of depression: population-level analysis of intervention cost-effectiveness in 14 world regions. Br J Psychiatry. 2004;184:393-403.

14. Dezetter A, Briffault X, Ben Lakhdar C, Kovess-Masfety V. Costs and benefits of improving access to psychotherapies for common mental disorders. The journal of mental health policy and economics. 2013;16(4):161-77.

15. Fantino B, Moore N, Verdoux H, Auray J-P. Cost-effectiveness of escitalopram vs. citalopram in major depressive disorder. Int Clin Psychopharmacol. 2007;22(2):107-15.

16. Gensichen J, Petersen JJ, Von Korff M, Heider D, Baron S, Konig J, et al. Cost-effectiveness of depression case management in small practices. British Journal of Psychiatry. 2013;202(6):441-6.

17. Gerhards SA, de Graaf LE, Jacobs LE, Severens JL, Huibers MJ, Arntz A, et al. Economic evaluation of online computerised cognitive-behavioural therapy without support for depression in primary care: randomised trial. Br J Psychiatry. 2010;196(4):310-8.

18. Gusi N, Reyes MC, Gonzalez-Guerrero JL, Herrera E, Garcia JM. Cost-utility of a walking programme for moderately depressed, obese, or overweight elderly women in primary care: a randomised controlled trial. BMC public health. 2008;8:231.

19. Haji EO, Mann K, Dragicevic A, Muller MJ, Boland K, Rao ML, et al. Potential cost-effectiveness of therapeutic drug monitoring for depressed patients treated with citalopram. Therapeutic Drug Monitoring. 2013;35(3):396-401.

20. Hollinghurst S, Carroll FE, Abel A, Campbell J, Garland A, Jerrom B, et al. Cost-effectiveness of cognitive-behavioural therapy as an adjunct to pharmacotherapy for treatment-resistant depression in primary care: economic evaluation of the CoBalT Trial. The British Journal of Psychiatry. 2014;204(1):69-76.

21. Hollinghurst S, Peters TJ, Kaur S, Wiles N, Lewis G, Kessler D. Cost-effectiveness of therapist-delivered online cognitive-behavioural therapy for depression: randomised controlled trial. Br J Psychiatry. 2010;197(4):297-304.

22. Humphreys I, Thomas S, Phillips C, Lincoln N. Cost analysis of the Communication and Low Mood (CALM) randomised trial of behavioural therapy for stroke patients with aphasia. Clinical Rehabilitation. 2015;29(1):30-41.

23 Jonkers CC, Lamers F, Evers SM, Bosma H, Metsemakers JF, Van Eijk JT. Economic evaluation of a minimal psychological intervention in chronically ill elderly patients with minor or mild to moderate depression: a randomized trial (the DELTA-study). International journal of technology assessment in health care. 2009;25(4):497-504.

24. Kafali N, Cook B, Canino G, Alegria M. Cost-effectiveness of a randomized trial to treat depression among Latinos. The journal of mental health policy and economics. 2014;17(2):41-50.

25 Katon W, Unutzer J, Fan MY, Williams JW, Jr., Schoenbaum M, Lin EH, et al. Cost-effectiveness and net benefit of enhanced treatment of depression for older adults with diabetes and depression. Diabetes Care. 2006;29(2):265-70.

26. Kendrick T, Peveler R, Longworth L, Baldwin D, Moore M, Chatwin J, et al. Cost-effectiveness and cost-utility of tricyclic antidepressants, selective serotonin reuptake inhibitors and lofepramine: Randomised controlled trial. British Journal of Psychiatry. 2006;188(APR.):337-45.

27. Klug G, Hermann G, Fuchs-Nieder B, Panzer M, Haider-Stipacek A, Zapotoczky HG, et al. Clinically and economically effectiveness of home treatment for elderly patients with depression: Randomised controlled trial. Journal of Mental Health Policy and Economics. 2011;14:S15.

28. Knapp M, King D, Romeo R, Schehl B, Barber J, Griffin M, et al. Cost effectiveness of a manual based coping strategy programme in promoting the mental health of family carers of people with dementia (the START (STrAtegies for RelaTives) study): a pragmatic randomised controlled trial. BMJ (Clinical research ed). 2013;347:f6342.

29. Lapierre Y, Bentkover J, Schainbaum S, Manners S. Direct cost of depression: analysis of treatment costs of paroxetine versus Imipramine in Canada. Canadian journal of psychiatry Revue canadienne de psychiatrie. 1995;40(7):370-7.

30. Liu C-F, Hedrick SC, Chaney EF, Heagerty P, Felker B, Hasenberg N, et al. Cost-effectiveness of collaborative care for depression in a primary care veteran population. Psychiatric Services. 2003;54(5):698-704.

31. Lokkerbol J, Adema D, Cuijpers P, Reynolds ICF, Schulz R, Weehuizen R, et al. Improving the cost-effectiveness of a healthcare system for depressive disorders by implementing telemedicine: A health economic modeling study. American Journal of Geriatric Psychiatry. 2014;22(3):253-62.

32. McCrone P, Knapp M, Proudfoot J, Ryden C, Cavanagh K, Shapiro DA, et al. Cost-effectiveness of computerised cognitive-behavioural therapy for anxiety and depression in primary care: randomised controlled trial. Br J Psychiatry. 2004;185:55-62.

33. McDonald WM, Phillips VL, Figiel GS, Marsteller FA, Simpson CD, Bailey MC. Cost-effective maintenance treatment of resistant geriatric depression. Psychiatric Annals. 1998;28(1):47-52.

34. Olgiati P, Bajo E, Bigelli M, De Ronchi D, Serretti A. Should pharmacogenetics be incorporated in major depression treatment? Economic evaluation in high- and middle-income European countries. Prog Neuropsychopharmacol Biol Psychiatry. 2012;36(1):147-54.

35. Olgiati P, Bajo E, Bigelli M, Montgomery S, Serretti A. Challenging sequential approach to treatment resistant depression: Cost-utility analysis based on the Sequenced Treatment Alternatives to Relieve Depression (STAR⁎D) trial. European Neuropsychopharmacology. 2013;23(12):1739-46.

36. Pyne JM, Fortney JC, Tripathi SP, Maciejewski ML, Edlund MJ, Williams DK. Cost-effectiveness analysis of a rural telemedicine collaborative care intervention for depression. Arch Gen Psychiatry. 2010;67(8):812-21.

37. Richards A, Barkham M, Cahill J, Richards D, Williams C, Heywood P. PHASE: a randomised, controlled trial of supervised self-help cognitive behavioural therapy in primary care. The British journal of general practice : the journal of the Royal College of General Practitioners. 2003;53(495):764-70.

38. Roijen LHV, Van Straten A, Maiwenn A, Rutten F, Donker M. Cost-utility of brief psychological treatment for depression and anxiety. British Journal of Psychiatry. 2006;188(APR.):323-9.

39. Romeo R, Knapp M, Banerjee S, Morris J, Baldwin R, Tarrier N, et al. Treatment and prevention of depression after surgery for hip fracture in older people: cost-effectiveness analysis. J Affect Disord. 2011;128(3):211-9.

40. Romeo R, Patel A, Knapp M, Thomas C. The cost-effectiveness of mirtazapine versus paroxetine in treating people with depression in primary care. Int Clin Psychopharmacol. 2004;19(3):125-34.

41. Rost K, Fortney J, Zhang M, Smith J, Smith GR, Jr. Treatment of depression in rural Arkansas: policy implications for improving care. The Journal of rural health : official journal of the American Rural Health Association and the National Rural Health Care Association. 1999;15(3):308-15.

42. Saylan M, Treur MJ, Postema R, Dilbaz N, Savas H, Heeg BM, et al. Cost-effectiveness analysis of aripiprazole augmentation treatment of patients with major depressive disorder compared to olanzapine and quetiapine augmentation in Turkey: A microsimulation approach. Value in Health Regional Issues. 2013;2(2):171-80.

43. Serrano-Blanco A, Gabarron E, Garcia-Bayo I, Soler-Vila M, Caramés E, Peñarrubia-Maria MT, et al. Effectiveness and cost-effectiveness of antidepressant treatment in primary health care: A six-month randomised study comparing fluoxetine to imipramine. J Affect Disord. 2006;91(2-3):153-63.

44. Serrano-Blanco A, Suárez D, Pinto-Meza A, Peñarrubia MT, Haro JM. Fluoxetine and imipramine: Are there differences in cost-utility for depression in primary care? Journal of evaluation in clinical practice. 2009;15(1):195-203.

45. Shah A, Wuntakal B, Fehler J, Sullivan P. Is a dedicated specialist social worker working exclusively with psychogeriatric inpatients and an associated dedicated domiciliary care package cost-effective? International Psychogeriatrics. 2001;13(3):337-46.

46 Shen C, Shah N, Findley PA, Sambamoorthi U. Depression treatment and short-term healthcare expenditures among elderly Medicare beneficiaries with chronic physical conditions. Journal of Negative Results in BioMedicine. 2013;12(1).

47. Sicras-Mainar A, Navarro-Artieda R, Blanca-Tamayo M, Gimeno-de la Fuente V, Salvatella-Pasant J. Comparison of escitalopram vs. citalopram and venlafaxine in the treatment of major depression in Spain: Clinical and economic consequences. Curr Med Res Opin. 2010;26(12):2757-64.

48. Simon GE, VonKorff M, Heiligenstein JH, Revicki DA, Grothaus L, Katon W, et al. Initial antidepressant choice in primary care: Effectiveness and cost of fluoxetine vs tricyclic antidepressants. Journal of the American Medical Association. 1996;275(24):1897-902.

49. Smit F, Willemse G, Koopmanschap M, Onrust S, Cuijpers P, Beekman A. Cost-effectiveness of preventing depression in primary care patients: randomised trial. Br J Psychiatry. 2006;188:330-6.

50. Stant AD, TenVergert EM, Kluiter H, Conradi HJ, Smit A, Ormel J. Cost-effectiveness of a psychoeducational relapse prevention program for depression in primary care. Journal of Mental Health Policy and Economics. 2009;12(4):195-204.

51. Titov N, Dear BF, Ali S, Zou JB, Lorian CN, Johnston L, et al. Clinical and Cost-Effectiveness of Therapist-Guided Internet-Delivered Cognitive Behavior Therapy for Older Adults With Symptoms of Depression: A Randomized Controlled Trial. Behavior Therapy. 2015;46(2):193-205.

52. Tournier M, Crott R, Gaudron Y, Verdoux H. Economic impact of antidepressant treatment duration in naturalistic conditions. Acta Psychiatr Scand. 2013;127(5):365-72. Tyrer P, Cooper S, Salkovskis P,

53. Tyrer H, Crawford M, Byford S, et al. Clinical and cost-effectiveness of cognitive behaviour therapy for health anxiety in medical patients: A multicentre randomised controlled trial. The Lancet. 2014;383(9913):219-25.

54. Unutzer J, Katon WJ, Fan MY, Schoenbaum MC, Lin EH, Della Penna RD, et al. Long-term cost effects of collaborative care for late-life depression. Am J Manag Care. 2008;14(2):95-100.

55. Wade AG, Saragoussi D, Despiegel N, Francois C, Guelfucci F, Toumi M. Healthcare expenditure in severely depressed patients treated with escitalopram, generic SSRIs or venlafaxine in the UK. Curr Med Res Opin. 2010;26(5):1161-70.

56. van den Berg M, Smit F, Vos T, van Baal PH. Cost-effectiveness of opportunistic screening and minimal contact psychotherapy to prevent depression in primary care patients. PLoS One. 2011;6(8):e22884.

57. Wiles N, Thomas L, Abel A, Barnes M, Carroll F, Ridgway N, et al. Clinical effectiveness and cost-effectiveness of cognitive behavioural therapy as an adjunct to pharmacotherapy for treatment-resistant depression in primary care: the CoBalT randomised controlled trial. Health Technol Assess. 2014;18(31):1-167, vii-viii.

58. Von Korff M, Katon W, Bush T, Lin EH, Simon GE, Saunders K, et al. Treatment costs, cost offset, and cost-effectiveness of collaborative management of depression. Psychosom Med. 1998;60(2):143-9.

**Intervention**

1. Banerjee S, Hellier J, Romeo R, Dewey M, Knapp M, Ballard C, et al. Study of the use of antidepressants for depression in dementia: The HTA-SADD trial- A multicentre, randomised, double-blind, placebo-controlled trial of the clinical effectiveness and cost-effectiveness of sertraline and mirtazapine. Health Technol Assess. 2013;17(7):1-43.

2. Jonkers CC, Lamers F, Evers SM, Bosma H, Van Eijk JT. Cost-utility estimates in depression: does the valuation method matter? The journal of mental health policy and economics. 2010;13(4):189-97.

3. Klug G, Hermann G, Fuchs-Nieder B, Panzer M, Haider-Stipacek A, Zapotoczky HG, et al. Effectiveness of home treatment for elderly people with depression: randomised controlled trial. Br J Psychiatry. 2010;197:463-7.

4. Markle-Reid M, McAiney C, Forbes D, Thabane L, Gibson M, Browne G, et al. An interprofessional nurse-led mental health promotion intervention for older home care clients with depressive symptoms. BMC Geriatr. 2014;14:62.

5. Parker SG, Oliver P, Pennington M, Bond J, Jagger C, Enderby PM, et al. Rehabilitation of older patients: day hospital compared with rehabilitation at home - a randomised controlled trial (Provisional abstract). Health Technol Assess [Internet]. 2009; (39):[i-xi pp.]. Available from: http://onlinelibrary.wiley.com/o/cochrane/cleed/articles/NHSEED-22009103815/frame.html.

6. Reynolds CF, 3rd, Cuijpers P, Patel V, Cohen A, Dias A, Chowdhary N, et al. Early intervention to reduce the global health and economic burden of major depression in older adults. Annual review of public health. 2012;33:123-35.

7. Sobocki P, Jonsson B, Angst J, Rehnberg C. Cost of depression in Europe. Journal of Mental Health Policy and Economics. 2006;9(2):87-98.

8. van der Weele GM, de Waal MW, van den Hout WB, van der Mast RC, de Craen AJ, Assendelft WJ, et al. Yield and costs of direct and stepped screening for depressive symptoms in subjects aged 75 years and over in general practice. Int J Geriatr Psychiatry. 2011;26(3):229-38.

9. van der Weele GM, de Waal MW, van den Hout WB, de Craen AJ, Spinhoven P, Stijnen T, et al. Effects of a stepped-care intervention programme among older subjects who screened positive for depressive symptoms in general practice: the PROMODE randomised controlled trial. Age Ageing. 2012;41(4):482-8.

Outcome

1. Aziz M, Mehringer AM, Mozurkewich E, Razik GN. Cost-utility of 2 maintenance treatments for older adults with depression who responded to a course of electroconvulsive therapy: results from a decision analytic model. Canadian journal of psychiatry Revue canadienne de psychiatrie. 2005;50(7):389-97.

2. Holland J, Bhogle M. Sertraline and mirtazapine as geriatric antidepressants. Psychiatria Danubina. 2013;25 Suppl 2:S286-90.

3. Shapira B, Tubi N, Drexler H, Lidsky D, Calev A, Lerer B. Cost and benefit in the choice of ECT schedule. Twice versus three times weekly ECT. Br J Psychiatry. 1998;172:44-8.

4. Shih YC, Bekele NB, Xu Y. Use of Bayesian net benefit regression model to examine the impact of generic drug entry on the cost effectiveness of selective serotonin reuptake inhibitors in elderly depressed patients. PharmacoEconomics. 2007;25(10):843-62.

5. Smith A, Graham L, Senthinathan S. Mindfulness-based cognitive therapy for recurring depression in older people: a qualitative study. Aging Ment Health. 2007;11(3):346-57.

6. Sobocki P, Ekman M, Agren H, Runeson B, Jonsson B. The mission is remission: Health economic consequences of achieving full remission with antidepressant treatment for depression. Int J Clin Pract. 2006;60(7):791-8.

7. Unützer J, Powers D, Katon W, Langston C. From establishing an evidence-based practice to implementation in real-world settings: IMPACT as a case study. Psychiatric Clinics of North America. 2005;28(4):1079-92.

8. Vasiliadis HM, Latimer E, Dionne PA, Preville M. The costs associated with antidepressant use in depression and anxiety in community living older adults. European Psychiatry. 2013;28.

9. Wiley-Exley E, Domino ME, Maxwell J, Levkoff SE. Cost-effectiveness of integrated care for elderly depressed patients in the PRISM-E study. The journal of mental health policy and economics. 2009;12(4):205-13.

10. Wu E, Greenberg P, Yang E, Yu A, Ben-Hamadi R, Erder MH. Comparison of treatment persistence, hospital utilization and costs among major depressive disorder geriatric patients treated with escitalopram versus other SSRI/SNRI antidepressants. Curr Med Res Opin. 2008;24(10):2805-13.

11. Wunner C, Reichhart C, Strauss B, Söllner W. Effectiveness of a psychosomatic day hospital treatment for the elderly: A naturalistic longitudinal study with waiting time before treatment as control condition. Journal of Psychosomatic Research. 2014;76(2):121-6.

12. Zimmer B. Direct and indirect costs of venlafaxine treatment of depression in the elderly with comorbid medical disorders. Annals of Long-Term Care. 1999;7(11):405-9.

Type of publication

1. Brettschneider C, Konig HH. Cost-utility analyses of cognitive-behavioral therapy for major depressive disorder - A systematic review. Value in Health. 2013;16(7):A549.

2. Johnson-Lawrence V, Kaplan G, Galea S. Socio-economic patterning in adulthood and depressive symptoms among a community sample of older adults in the United States. Public Health. 2015.

3. Ladapo JA, Shaffer JA, Fang Y, Ye S, Davidson KW. Cost-effectiveness of enhanced depression care after acute coronary syndrome: results from the Coronary Psychosocial Evaluation Studies randomized controlled trial. Archives of internal medicine. 2012;172(21):1682-4.

4. Meyers BS. Optimizing the use of data generated by geriatric depression treatment studies during a time of diminishing resources. Am J Geriatr Psychiatry. 2007;15(7):545-52.

5. Schapira M, Smietniansky M, Patino H, Castejon G, Outumuro MB, Esteban J, et al. Medical services use by ambulatory elderly with depression and anxiety. A collaborative care management program. J Am Geriatr Soc. 2013;61:S59.

6. Simon G. In treatment-resistant depression, adding cognitive-behavioral therapy to usual care was cost-effective at 1 y. Annals of Internal Medicine. 2014;160(12):JC13.
